# Hepatic mitochondrial signaling as a systemic hub: inter-organ communication networks in aging and aging-related diseases

**DOI:** 10.3389/fcell.2026.1745201

**Published:** 2026-02-12

**Authors:** Yishuo Ji, Ying Wang, Xiaowei Yu, Yi Jin, Kai Zhao, Yue Hu, Zhenglin He

**Affiliations:** 1 Department of Biobank, China-Japan Union Hospital of Jilin University, Changchun, China; 2 The First Bethune Hospital of Jilin University, Jilin University, Changchun, China; 3 School of Cellular and Molecular Medicine, University of Bristol, Bristol, United Kingdom; 4 Department of Immunobiology, Yale University School of Medicine, New Haven, CT, United States; 5 Center of Molecular and Cellular Oncology, Yale Cancer Center, Yale University, New Haven, CT, United States

**Keywords:** aging, diseases, hepatic mitochondria, inter-organ communication, mitokines, mtDNA, mtROS, UPRmt

## Abstract

Aging and aging-related diseases are increasingly viewed as systemic disorders arising from disrupted inter-organ communication, yet the mechanisms linking local metabolic stress to organism-wide dysfunction remain unclear. The liver occupies a central position in this network, but how hepatic mitochondrial stress is translated into circulating signals that remodel distant tissues is incompletely understood. Here, we synthesize evidence identifying hepatic mitochondria as a systemic signaling hub that integrates metabolic and inflammatory stress and disseminates blood-borne cues during aging. We focus on three major classes of mitochondrial outputs: UPRmt-driven mitokines, including fibroblast growth factor 21 (FGF21) and growth differentiation factor 15 (GDF15); metabolites generated through mitochondrial metabolic reprogramming; and mitochondrial danger signals such as mitochondrial reactive oxygen species (mtROS) and oxidized mitochondrial DNA (mtDNA). These signals act through endocrine, metabolic, and immune pathways to reshape mitochondrial function, inflammation, and energy homeostasis across multiple organs. We further discuss how aging shifts hepatic mitochondrial signaling from adaptive to maladaptive states and emphasize that liver-centered regulation operates within bidirectional networks involving the gut, skeletal muscle, and immune system. Finally, we outline translational challenges and potential strategies for modulating hepatic mitochondrial outputs to restore systemic homeostasis in aging and aging-related diseases.

## 1 Introduction

Recent studies have demonstrated that local stress in metabolic organs such as the liver, adipose tissue, or muscle can remotely reprogram the function and fate of distant tissues through circulating factors (Bar-Ziv et al., 2020). Therefore, elucidating inter-organ communication not only unveils the regulatory logic underlying physiological homeostasis but also provides a unifying theoretical framework for understanding aging-related multi-organ pathological conditions. Compared with tissue biopsy, blood sampling is minimally invasive; moreover, the short half-life of circulating signals allows for dynamic monitoring (Burtscher et al., 2023). More importantly, the “nutrient-sensing” nature of inter-organ communication makes dietary, exercise, and rehabilitation interventions more acceptable to the general population, aligning with the ultimate medical goal of prevention-first healthcare (Zhang B. et al., 2024). However, a systematic understanding of the entire inter-organ communication axis, encompassing signal origin, molecular mediators, and target reception, remains incomplete, which currently limits its clinical translation.

The liver serves as the central hub of systemic metabolism, playing a pivotal role in maintaining the homeostasis of carbohydrates, lipids, and amino acids (Van Laar et al., 2023; Abulikemu et al., 2023). All nutrients, microbial metabolites, and environmental toxins absorbed from the intestine first enter the portal circulation, where they undergo oxidative and reductive processing by hepatic mitochondria (Bar-Ziv et al., 2020; Fu et al., 2022). It is noteworthy that the liver functions not only as a metabolic center but also as a key node in inter-organ communication. Within this framework, hepatic mitochondria act as an integrative platform for metabolism, energy, and signaling. Through structural remodeling and functional adaptation, they enable metabolic reprogramming, energy output, and systemic signal transduction, thereby orchestrating organismal stress responses and whole-body homeostatic regulation (Fu et al., 2022; Chen et al., 2023; Zhang et al., 2021). In particular, mitochondrial quality control (MQC) and the UPRmt enable the liver to sense endogenous metabolic stress or mitochondrial damage, as well as exogenous cues such as nutrients, toxins, and microbial metabolites, and to translate these stimuli into cross-tissue molecular communication (Sohn et al., 2023; Yi, 2019). Consequently, hepatic mitochondria function as a natural compiler of circulating “communication molecules,” whose signaling outputs can reach skeletal muscle, adipose tissue, the brain, and the heart to remotely modulate MQC and energy metabolism (Zhang B. et al., 2024), thereby influencing the function and aging trajectory of distal organs.

**FIGURE 1:**
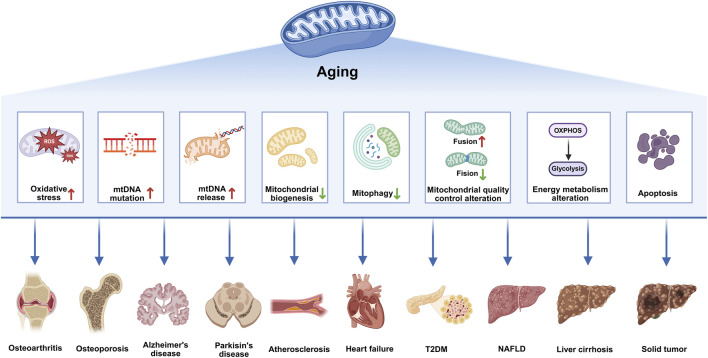
Mitochondrial dysfunction as a central driver of aging-related diseases. With aging, an increase in ROS production in mitochondria leads to oxidative stress, causing oxidative damage to DNA (especially mtDNA), lipids, and proteins. An increased mtDNA mutation rate causes increased frequencies of errors or mutations in mtDNA-encoded enzyme subunits, resulting in impaired OXPHOS. mtDNA is released into the cytoplasm or outside the cell and participates in SASP secretion by activating cGAS-STING pathways. Decreased mitophagy mediated by the PINK1/parkin ubiquitin pathway results in impaired clearance of damaged mitochondria. Reduced mitochondrial biogenesis mediated by PGC1 and NRF decreases the number of newborn mitochondria. During aging, mitochondria show altered quality control changes, Drp1/FIS1-mediated mitochondrial fission decreases, and MFN/OPA-mediated mitochondrial fusion increases, affecting mitochondrial shape and function. The mitophagy defects and mitochondrial dysfunction trigger Aβ and tau accumulation, leading to synaptic dysfunction and cognitive deficits during AD development. The metabolic transition from OXPHOS to glycolysis leads to altered metabolite generation. Mitochondrial pathway-mediated apoptosis is an important form of cell death. Mitochondrial dysfunction contributes to osteoarthritis, osteoporosis, Alzheimer’s disease, Parkinson’s disease, atherosclerosis, heart failure, T2DM, NAFLD, liver cirrhosis, and solid tumors by inducing oxidative stress, inflammation, apoptosis, and metabolic alterations. Abbreviations: amyloid-β, Aβ; Alzheimer’s disease, AD; cyclic GMP-AMP synthase-stimulator of interferon genes, cGAS-STING; dynamin-related protein 1, Drp1; mitochondrial fission 1 protein, FIS1; heart failure, HF; mitofusin, MFN; mitochondrial DNA, mtDNA; non-alcoholic fatty liver disease, NAFLD; nuclear respiratory factor, NRF; optic atrophy protein, OPA; oxidative phosphorylation, OXPHOS; peroxisome proliferator-activated receptor gamma coactivator 1-alpha, PGC1; PTEN-induced putative kinase 1, PINK1; reactive oxygen species, ROS; senescence-associated secretory phenotype, SASP; type 2 diabetes mellitus, T2DM.

Existing evidence indicates that the hallmarks of aging serve as shared drivers of aging-related diseases. However, aging-related diseases across different organs and systems are characterized by distinct combinations of these molecular hallmarks (Guo et al., 2022). Mitochondrial dysfunction is one of the most common causes of various aging-related diseases (Figure 1). The essence of organismal aging lies in the progressive breakdown of inter-organ homeostatic coordination rather than the decline of individual organ function (Oh et al., 2023). The accumulation of senescent cells and the persistent release of inflammatory and stress-associated signals further distort intercellular communication, leading to maladaptive feedback between organs. As a result, aging emerges as a systems-level process driven by the breakdown of integrated organ-to-organ coordination, which ultimately makes multiple tissues more susceptible to dysfunction (Gulej et al., 2025). The liver and its mitochondria not only determine systemic metabolic homeostasis but also act as amplifiers and central hubs of inter-tissue communication, exerting global regulatory effects during aging and related pathologies. Aging are accompanied by attenuated or dysregulated hepatic UPRmt activation and disruption of the mitokine profile, leading to the replacement of “youth-protective signals” with “aging-amplifying signals,” thereby driving the functional decline of multiple organs (Patel et al., 2022). However, the spatiotemporal dynamics of this “liver-blood-multi-organ” communication axis remain insufficiently characterized in aging and aging-related diseases, and critical questions such as its reversibility and safe intervention strategies remain unresolved.

**FIGURE 2:**
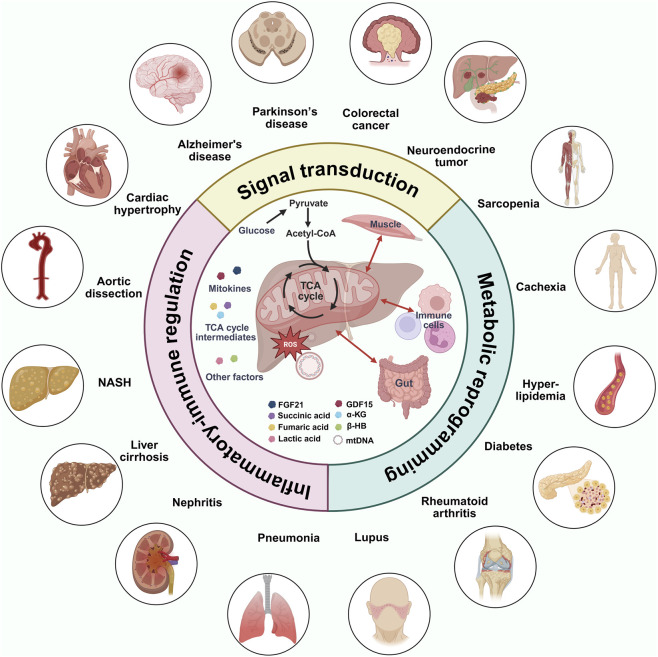
Liver mitochondrial signaling as a systemic hub coordinating inter-organ communication across aging and aging-related diseases contexts. This schematic depicts hepatic mitochondria as a central hub that integrates metabolic stress and disseminates circulating signals to distal organs, highlighting three major functional dimensions: signal transduction, metabolic reprogramming, and inflammatory-immune regulation. Liver-derived mitochondrial signals include UPRmt-associated factors such as FGF21 and GDF15; metabolic reprogramming mediators such as succinate, fumarate, α-KG, β-HB, and lactate, as well as mtDNA and mtROS. Through these signals, the liver engages in bidirectional inter-organ communication with skeletal muscle, the gut, and immune cells via the circulation. Beyond local crosstalk, these mitochondria-derived factors exert systemic effects that have been implicated in a broad spectrum of diseases, including cardiovascular disorders such as cardiac hypertrophy and aortic dissection, autoimmune diseases such as systemic lupus erythematosus and rheumatoid arthritis, and neurodegenerative diseases including Alzheimer’s disease and Parkinson’s disease and so on. Abbreviations: growth differentiation factor 15, GDF15; fibroblast growth factor 21, FGF21; α-ketoglutarate, α-KG; β-hydroxybutyrate, β-HB; mitochondrial reactive oxygen species, mtROS; mitochondrial DNA, mtDNA; tricarboxylic acid, TCA; non-alcoholic steatohepatitis, NASH; coenzyme A, CoA.

This review focuses on hepatic mitochondria and systematically analyzes inter-organ communication networks mediated by three major mechanisms, including signal transduction, metabolic reprogramming, and inflammatory-immune regulation, with particular emphasis on their central roles in aging and aging-related diseases (Figure 2). By evaluating the translational bottlenecks of current therapeutic strategies, we propose innovative approaches that reprogram hepatic mitochondrial outputs via nutritional or pharmacological interventions, thereby providing a theoretical basis for integrating hepatic mitochondrial regulation into broad-spectrum anti-aging research. While we use aging as an organizing framework to illustrate progressive dysregulation of hepatic mitochondrial outputs, the mechanisms discussed, including UPRmt-driven mitokine release, metabolic reprogramming, and mtDNA-mediated inflammation, also operate under acute metabolic stress, toxin exposure, and obesity. In this sense, aging serves as a lens that exposes these pathways in a chronic and maladaptive state, providing insights that are broadly transferable across pathological contexts.

## 2 UPRmt-driven cross-tissue signaling under mitochondrial stress

During aging and aging-related diseases, hepatic mitochondrial function progressively declines, accompanied by reduced efficiency of MQC systems, including attenuated or dysregulated UPRmt activation. This impairment not only disrupts hepatic function itself but also accelerates aging and functional decline in distal tissues through aberrant signaling outputs (López-Otín et al., 2023).

As the cellular powerhouses and metabolic centers, mitochondria rely on the precise regulation of mitochondrial protein homeostasis to maintain proper function (Konovalova et al., 2023). Upon exposure to various stressors such as oxidative stress or the accumulation of misfolded proteins, cells activate the UPRmt to cope with the crisis (Shi et al., 2024; Xu et al., 2023). The core mission of the UPRmt is to sense imbalances in mitochondrial proteostasis and to initiate specific transcriptional programs that enhance protein folding and degradation capacity, thereby restoring mitochondrial homeostasis under stress conditions. The most well-characterized signaling cascade of this response is the activating transcription factor 5 (ATF5)/activating transcription factor associated with stress-1 (ATFS-1)-dependent pathway (Liu Y. et al., 2023). Under physiological conditions, ATFS-1 is imported into mitochondria and rapidly degraded via the proteolytic pathway, thereby maintaining a low basal level (Dodge et al., 2024). When mitochondrial stress occurs, such as a loss of membrane potential or the accumulation of unfolded proteins, the import efficiency mediated by the mitochondrial targeting sequence (MTS) peptide decreases, and ATFS-1 instead utilizes its nuclear localization signal (NLS) to translocate into the nucleus (Zhang X. et al., 2024). Within the nucleus, ATFS-1 directly binds to the promoter regions of UPRmt target genes, activating the transcription of a wide range of genes, including molecular chaperones (e.g., HSP60), proteases (e.g., ClpP), and numerous metabolic regulators (Torres et al., 2024). In mammals, ATF5 is regarded as the functional ortholog of ATFS-1. In response to mitochondrial proteostatic imbalance, ATF5 similarly accumulates in the nucleus, inducing the expression of genes encoding mitochondrial chaperones and proteases (Katiyar et al., 2020), thereby enhancing the protein folding and degradation capacity to effectively restore mitochondrial functional homeostasis.

In addition to the ATF5-mediated direct transcriptional response, mitochondrial stress can also activate the activating transcription factor 4 (ATF4)-C/EBP homologous protein (CHOP) axis through the integrated stress response (ISR), constituting an alternative regulatory branch of the UPRmt (Quirós et al., 2016). During this process, ISR-associated kinases such as GCN2 and PERK phosphorylate eIF2α (Pontanari et al., 2025), thereby globally suppressing protein synthesis to reduce the mitochondrial folding load, while selectively enhancing ATF4 translation (Dang et al., 2023); Activated ATF4 translocates into the nucleus to induce CHOP expression, which subsequently forms a heterodimer with C/EBPβ to promote the transcription of UPRmt target genes such as HSP60, HSP10, and ClpP (Wu et al., 2024). At the same time, ATF4/CHOP, as an upstream regulator, promotes the expression of ATF5 or activates its function, and ultimately enhances the quality control of mitochondrial proteins and alleviates stress through this cascade regulation (Zhou et al., 2022). Notably, these signaling axes are not entirely independent but instead exhibit extensive cross-regulation and signal integration; for instance, HSF1 cooperates with ATF5 to potentiate CHOP activity (Katiyar et al., 2020). Impaired mitochondrial protein import—such as that caused by Tim23 deficiency—results in cytosolic accumulation of mitochondrial precursor proteins (c-mtProt), which triggers eIF2α phosphorylation and subsequent activation of ATF5 and its downstream pathways (Oliveira and Hood, 2018). HSP60/70 can increase or decrease the affinity or binding ratio of interaction with CHOP protein under mild and severe stress conditions, so as to regulate the nuclear translocation level of CHOP protein and realize the regulation of stress degree (Cozzolino et al., 2023). Through this coordinated enhancement of protein folding and clearance capacities, UPRmt effectively restores intramitochondrial proteostasis, ensures proper assembly of functional complexes such as oxidative phosphorylation (OXPHOS), stabilizes mitochondrial performance, and ultimately improves cellular survival under stress conditions.

With advancing age and in aging-related diseases, hepatic UPRmt signaling progressively loses its adaptive precision, characterized by delayed induction, attenuated amplitude, and prolonged or inappropriate activation kinetics (López-Otín et al., 2023). This functional deterioration not only compromises the liver’s intrinsic capacity to manage mitochondrial stress but, more importantly, impairs its role as a central signaling hub, disrupting its ability to accurately sense, integrate, and transmit inter-organ communication cues (Sohn et al., 2023; López-Otín et al., 2023). Moreover, several liver pathologies closely associated with aging, such as metabolic dysregulation, toxin exposure, and hepatic steatosis readily trigger UPRmt activation, thereby reinforcing a complex vicious cycle (Qi et al., 2023; Samuel and Shulman, 2012; Zhao et al., 2022).

Since the pioneering discovery by Durieux et al. that neuron-specific activation of the UPRmt in *C. elegans* can remotely enhance intestinal mitochondrial function and extend lifespan (Durieux et al., 2011), extensive research has explored the inter-organ regulatory mechanisms of UPRmt. Activation of hepatic UPRmt, for instance, has been shown to influence distant tissues such as the intestine, skeletal muscle, and even tumor progression (Zhang B. et al., 2024; Liu Y. et al., 2022; Woytash et al., 2025), primarily through the secretion of specific circulating factors that mediate systemic communication. These stress-induced secreted signaling molecules, released in response to mitochondrial perturbations, particularly UPRmt activation, and capable of exerting systemic effects, are collectively referred to as mitokines. As canonical outputs of the UPRmt, mitokines represent a pivotal transition point where mitochondrial stress signaling extends beyond organelle-level homeostasis to orchestrate whole-body metabolic regulation. Among the mitokines identified to date, the downstream mechanisms of GDF15 and FGF21 have been most extensively characterized. Beyond these classical mitokines, UPRmt-mediated inter-organ communication can also occur through the release of mitochondrial components such as mtDNA, or through metabolic reprogramming signals including α-ketoglutarate (α-KG) (Burtscher et al., 2023).

### 2.1 FGF21

FGF21 belongs to the fibroblast growth factor (FGF) family but differs from most FGFs in that it functions as an endocrine factor, circulating through the bloodstream to act on distal target organs and thereby playing a central role in systemic metabolic regulation (Kaur et al., 2022). In this regard, the liver serves as the primary source of circulating FGF21 (Gao et al., 2019). Emerging evidence indicates that activation of the UPRmt in the liver acts as a key upstream mechanism controlling FGF21 expression and secretion, with the ATF4 signaling axis serving as the central pathway mediating this induction (Yuan et al., 2025). Various stimuli that trigger UPRmt activation, particularly amino acid deprivation such as leucine, cysteine, or methionine deficiency, activate key branches of the ISR-UPRmt signaling network, including GCN2-mediated eIF2α phosphorylation, thereby leading to elevated ATF4 protein levels (Jonsson et al., 2022; Stone et al., 2021; Varghese et al., 2025). Elevated ATF4 subsequently drives robust transcriptional upregulation of FGF21. Experimental evidence demonstrates that inhibition of the PERK-ATF4 signaling pathway effectively abolishes stress-induced elevation of FGF21 expression (Wang et al., 2018). Mitochondrial stress states associated with enhanced fatty acid oxidation can likewise augment hepatic FGF21 expression through activation of ATF-family signaling pathways, including ATF4 and its related transcriptional regulators (Jena et al., 2023; Hondares et al., 2010).

The elevation of hepatic FGF21 expression exerts both beneficial and detrimental effects (Fisher and Maratos-Flier, 2016; De Sousa-Coelho et al., 2023). Analogous to the transient therapeutic effects of cardiac glycosides used in acute and chronic heart failure, short-term elevation of FGF21 acts primarily on adipose tissue to enhance glucose uptake and lipolysis, modulates hypothalamic energy balance and neuroendocrine output, and promotes hepatic fatty acid oxidation and ketogenesis (Szczepańska and Gietka-Czernel, 2022; Prida et al., 2022). Collectively, these actions confer clear metabolic benefits, including systemic insulin sensitization and body weight reduction (Corbee et al., 2022). Recent studies in Parkinson’s disease models have further demonstrated that liver-derived FGF21 can activate the adenosine monophosphate-activated protein kinase (AMPK)/peroxisome proliferator-activated receptor gamma coactivator 1-alpha (PGC-1α) signaling pathway in the brain to restore mitochondrial integrity and suppress neuroinflammation (Fang et al., 2020). In cardiomyocytes, FGF21 engages β-klotho (KLB) and fibroblast growth factor receptor (FGFR) to form a functional trimeric complex that mitigates oxidative stress, pathological hypertrophy, and lipotoxic injury (Kaur et al., 2023). Through these mechanisms, FGF21 confers both neuroprotective and cardioprotective effects.

However, chronic elevation of FGF21 is frequently accompanied by “FGF21 resistance” characterized by reduced responsiveness of target tissues that diminishes its protective effects (Keipert and Ost, 2021). For instance, expression of KLB in white adipose tissue (WAT), brown adipose tissue (BAT), skeletal muscle, the pancreas, and specific hypothalamic regions is markedly decreased in models of obesity or chronic mitochondrial stress. Consequently, despite persistently high circulating levels of FGF21, its metabolic and protective actions are largely blunted. Moreover, accumulating evidence suggests that sustained elevation of FGF21 may confer pro-tumorigenic risk (Qiu et al., 2025). Sui and colleagues demonstrated that FGF21 expression is upregulated in several cancers, including colorectal carcinoma, melanoma, and breast cancer (Sui and Chen, 2022). Elevated FGF21 levels correlated positively with tumor volume and were predictive of poor prognosis, such as reduced survival in patients with colorectal cancer. Mechanistically, Hu et al.’s work revealed that FGF21 promotes tumor progression by suppressing antitumor immunity through the reprogramming of cholesterol metabolism in CD8^+^ T cells (Qiu et al., 2025; Hu et al., 2024). Furthermore, targeting tumor-derived FGF21 has been reported to attenuate cachexia in experimental colorectal cancer models (Pin et al., 2023). Collectively, these findings indicate that chronic FGF21 signaling shifts from metabolic protection to pathological effects, highlighting FGF21 as a context-dependent target in aging, cancer, and aging-related diseases.

### 2.2 GDF15

GDF15, a member of the transforming growth factor-β (TGF-β) superfamily, is normally expressed at low constitutive levels in tissues such as the placenta, kidney, and liver (Wan and Fu, 2024). However, its expression is markedly induced under various stress conditions, including cellular injury, inflammation, hypoxia, and metabolic disturbances (Salminen, 2024). For instance, cardiomyocytes subjected to ischemic injury and hepatocytes exposed to toxic insults both release substantial amounts of GDF15 (Sigvardsen et al., 2025).

Activation of the hepatic UPRmt represents a key upstream mechanism driving GDF15 expression and secretion, involving multiple signaling pathways. Tang et al. (2024) found that chemotherapy drugs activate the inositol-requiring enzyme 1 alpha (IRE1 α)-X-box binding protein 1 (XBP1) signaling pathway in the liver to produce spliced XBP1 (XBP1s). XBP1s can directly bind to the ERSE element in the promoter region of the GDF15 gene, thereby positively regulating the expression and secretion of GDF15. When GDF15 in circulation reaches the brainstem, it will bind to GFRAL on the surface of neurons in the nucleus tractus solitarius and the caudal nucleus, thereby causing weight loss and anorexia in mice. Furthermore, Jena et al. (2023) revealed that the activation of UPRmt in the liver can also indirectly upregulate the expression and secretion of GDF15 by inducing eIF2α phosphorylation and ATF4 translation. The GDF15 that enters the bloodstream, in addition to its aforementioned effects on the brainstem, can also promote the conversion of WAT throughout the body to BAT, increase energy consumption, and enhance the clearance of fat in tissues such as muscles, thereby improving insulin sensitivity. Although Chung et al. demonstrated that muscle specific deletion of the mitochondrial ribosomal protein Crif1 activates the UPRmt and increases GDF15 secretion in skeletal muscle (Chung et al., 2017), the established roles of GDF15 across liver, muscle, and adipose tissue suggest that a comparable UPRmt-GDF15 regulatory axis may also operate in the liver.

The increased expression of GDF15 in liver is a double-edged sword. On the one hand, the elevated GDF15 is a physiological or moderate stress response, which may help with weight loss, improve metabolism and delay aging and aging-related diseases (Jena et al., 2023). On the other hand, high levels of GDF15 are often associated with aging and metabolic pathological conditions, predicting of disease severity and mortality (Salminen, 2024; Reyes et al., 2024; Ling et al., 2023; Du et al., 2025). Together, these opposing effects make GDF15 a promising mechanistic target for understanding and modulating aging-related diseases.

### 2.3 Other factors

Novel evidence suggests that liver mitochondrial stress or UPRmt may also affect the release of other secretory factors, such as FGF1 and interleukin-6 (IL-6) (Eigner et al., 2017; Xu et al., 2021). FGF1 is involved in metabolic regulation, while elevated IL-6 is associated with muscle/neural inflammation. However, it is still uncertain whether these factors are truly “mitokines”. There is a lack of direct regulatory evidence from UPRmt transcription factors for their responses, and their multi-organ secretion characteristics are difficult to trace back to liver-specific stress. Therefore, the mechanism study of these factors as cross-tissue communication mediators of mitochondria is far from reaching the research level of GDF15/FGF21.

**FIGURE 3:**
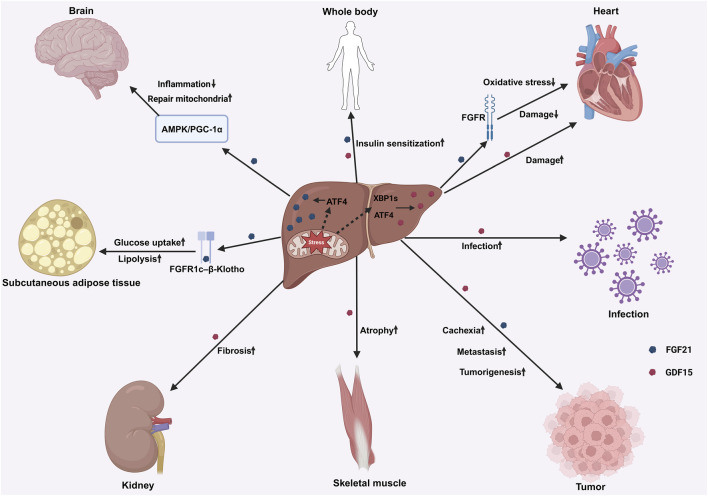
The regulatory effect and molecular mechanism of liver derived FGF21 and GDF15 on multiple organ systems of the whole body through inter-tissue communications. This schematic diagram highlights the interaction of two cytokines with brain, heart, kidney, skeletal muscle, subcutaneous adipose tissue and other organs through specific signaling pathways (such as FGF21-FGFR1c-β-Klotho pathway, GDF15 related pathway) in response to pathophysiological stimuli such as injury, stress, infection and so on. Abbreviations: growth differentiation factor 15, GDF15; fibroblast growth factor 21, FGF21; adenosine monophosphate-activated protein kinase, AMPK; peroxisome proliferator-activated receptor gamma coactivator 1-alpha, PGC-1α; fibroblast growth factor receptor, FGFR; activating transcription factor 4, ATF4; X-box binding protein 1, XBP1.

In conclusion, although liver mitochondrial stress may affect various secretory factors such as FGF1 and IL-6, there is no direct evidence yet as to whether they belong to “mitokines”, and the secretion sources are multi-organized, lacking liver specificity. Currently, the research on liver-derived UPRmt output signals mainly focuses on FGF21 and GDF15 (Figure 3). In the future, it is necessary to further clarify the expression changes of these factors under different stress conditions to comprehensively reveal the role of liver UPRmt in cross-tissue communication, metabolic homeostasis, and related diseases.

## 3 Inter-organ crosstalk in metabolic reprogramming

As the metabolic hub, the liver relies heavily on mitochondria to drive key pathways, including adenosine triphosphate (ATP) production, fatty acid β-oxidation, ketone body synthesis, the urea cycle, and one-carbon metabolism (Parlakgül et al., 2024), thereby sustaining systemic metabolic homeostasis. However, under pathological conditions such as aging and metabolic diseases, sustained metabolic stress (Horn and Tacke, 2024) drives the liver to initiate an adaptive strategy, namely, metabolic reprogramming. This is characterized by systemic alterations in metabolites, fluxes, and enzyme activities (Horn and Tacke, 2024), and represents a dynamic rebalancing of cellular energy demands, nutrient availability, and environmental stressors (Lin et al., 2024). Mitochondria serve as the central executors and signaling hubs of this process. On one hand, as executors of metabolic pathways, mitochondria directly regulate pathways such as the oxidative phosphorylation/glycolysis balance (Jin et al., 2020), they also integrate signals via mitochondrial-associated membranes (MAMs), calcium signaling, and dynamic changes to achieve global intracellular metabolic reprogramming (Morio et al., 2021). On the other hand, key mitochondrial metabolic intermediates, such as acetyl-CoA, α-KG, and succinate, act as “metabolite messengers” themselves (Jin et al., 2020; Morio et al., 2021). They participate in the regulation of intracellular signaling pathways (e.g., hypoxia-inducible factor 1 alpha (HIF-1α), mechanistic target of rapamycin (mTOR), and AMPK and epigenetics (Azzimato et al., 2021; Villanueva-Carmona et al., 2023; Sengupta et al., 2010), and are also closely associated with inter-organ crosstalk.

### 3.1 TCA cycle intermediates

In contexts that trigger hepatic metabolic reprogramming, such as the hypoxic microenvironment in nonalcoholic fatty liver disease (NAFLD) or the Warburg effect in cancer, mitochondrial TCA cycle homeostasis is disrupted in the liver (Yang et al., 2024; Baulies et al., 2018). Metabolites within the TCA cycle, including succinate, fumarate, and α-KG, transcend their roles as mere metabolites and act as key pathological molecules in intercellular signaling pathways (Marsal-Beltran et al., 2023).

#### 3.1.1 Succinic acid

Aberrant succinate accumulation is primarily driven by two mechanisms: suppression of succinate dehydrogenase (SDH) activity by a hypoxic microenvironment or the Warburg effect (Ricci et al., 2023), and indirect inhibition of mitochondrial SDH activity by isocitrate dehydrogenase 1 and 2 (IDH1/2) mutations (Lee et al., 2024). Among these, mechanisms related to IDH mutations have been extensively studied in neurological disorders but remain less explored in the liver (Lee et al., 2024). In contrast, the hypoxic microenvironment or Warburg effect in liver diseases (e.g., chronic liver injury, NAFLD, hepatocellular carcinoma (HCC) has been well investigated (Azzimato et al., 2021; Ricci et al., 2023). Specifically, the hypoxic environment in chronic liver injury induces the arrest of the TCA cycle in hepatic macrophages at the citrate and succinate stages, along with its uncoupling from oxidative phosphorylation—impeding succinate metabolism. Concurrently, hypoxia stabilizes HIF-1α, further promoting its accumulation (Azzimato et al., 2021). In HCC, loss of expression or impaired activity of SDHA/B is a key contributor to succinate accumulation (Yuan et al., 2023). Succinate accumulated in the liver “spills over” to form hypersuccinic acidemia. Its entry into the circulatory system occurs via two routes: active efflux through SLC13 transporters (e.g., SLC13A3) (Sampson et al., 2020), and more importantly, long-range delivery via exosomal encapsulation—allowing it to act as an intercellular signaling molecule (Liu C. et al., 2023). As a damage-associated molecular pattern (DAMP), circulating succinate primarily acts on the widely expressed succinate receptor 1 (SUCNR1) (Villanueva-Carmona et al., 2023; Rawal et al., 2024; Sabadell-Basallote et al., 2024). In macrophages, it binds to SUCNR1 to induce a proinflammatory phenotype and activate inflammasomes for interleukin-1 beta (IL-1β) release (Rawal et al., 2024). In adipocytes, it regulates BMAL1 and C/EBPα via the SUCNR1-AMPK/JNK pathway to inhibit leptin expression (Villanueva-Carmona et al., 2023). In pancreatic islet cells, it stimulates insulin secretion through the Gq-protein kinase C (PKC) pathway (Sabadell-Basallote et al., 2024). The danger of this liver-derived succinate overflow lies in its multi-organ targeting effect. For instance, in the progression of NAFLD to nonalcoholic steatohepatitis (NASH), it simultaneously exacerbates intrahepatic inflammation, adipose tissue insulin resistance, and β-cell dysfunction (Fernández-Veledo et al., 2024), thereby establishing a pathological positive feedback loop that amplifies disease progression across organs. Additionally, abnormalities in target organs further intensify liver-derived succinate overflow, creating a self-sustaining vicious cycle (Mills et al., 2021).

#### 3.1.2 Fumaric acid

When the liver is exposed to stress conditions such as NASH or toxicant exposure (Bacil et al., 2023; Sinton et al., 2019), mitochondrial homeostasis in the liver is disrupted, leading to fumarate accumulation in a manner analogous to succinate accumulation. Unlike the mechanism underlying elevated fumarate in patients with hereditary leiomyomatosis and renal cell carcinoma (HLRCC), the primary mechanism under stress conditions is TCA cycle dysregulation (Valcarcel-Jimenez and Frezza, 2023). The liver induces aberrant fumarate accumulation by suppressing fumarate hydratase (FH) activity and enhancing “reverse flux” in the TCA cycle (Luo et al., 2024). Accumulated fumarate further inhibits α-KG-dependent dioxygenases, disrupting HIF-1α stability and epigenetic regulation, which contributes to a vicious cycle involving oxidative stress, fumarate accumulation, and metabolic-epigenetic dysregulation, exacerbating liver injury and NASH progression (Sinton et al., 2019), Additionally, abnormal SDH function leads to the same outcome (Wang et al., 2021). Aberrantly accumulated fumarate increases the overall level of protein succinylation in the liver by competitively inhibiting desuccinylase activity and conversely elevating succinate concentration (Khidr et al., 2023), due to the abnormal function of their metabolic enzymes, these succinylated proteins cause cells to secrete specific metabolites (e.g., lactate) into the extracellular space, which then spread systemically via the circulation. Besides, these metabolites may be released extracellularly via extracellular vesicles (e.g., exosomes) and transported to other tissues through body fluids (blood, lymph) (Kalluri and LeBleu, 2020). However, the exosome-mediated pathway has not been confirmed to date (Vagner et al., 2018). Nevertheless, recent studies by Khidr et al. indicate that fumarate can indirectly regulate exosome release by succinylating Parkin, thereby altering membrane lipid composition or cellular energy metabolism in rotenone-induced Parkinson’s diseases in a rat model (Khidr et al., 2023; Cheng et al., 2022). In parallel, recent work from Lu et al. has shown that succinate-containing vesicles (SMPs) are delivered to tumor-associated macrophages via exosomes, leading to metabolic reprogramming (Lu et al., 2025). Together, these findings support the reasonable hypothesis that fumarate and succinate may modulate intercellular communication through vesicle-mediated pathways. Notably, circulating fumarate can act as a systemic signal, engaging defined stress and inflammatory pathways in distant tissues. Aberrantly elevated circulating fumarate stabilizes HIF-1α by inhibiting prolyl hydroxylases (PHDs), activates target genes such as ADAM17, and ultimately triggers systemic inflammation and vascular structural damage in aortic dissection (Lian et al., 2019). Furthermore, circulating fumarate acts as a DAMP to activate inflammatory pathways. For instance, fumarate and its derivatives (e.g., dimethyl fumarate and monomethyl fumarate) primarily activate downstream target genes via the nuclear factor erythroid 2-related factor 2 (NRF2) - kelch-like ECH-associated protein 1 (KEAP1) signaling pathway, leading to disrupted immune homeostasis, metabolic disturbances, and cytotoxicity (Hoyle et al., 2022). Intriguingly, Song et al. reached contradictory conclusions: they found that elevated circulating fumarate and dimethyl fumarate (DMF) activate downstream target genes (e.g., HO-1) via the Keap1-Nrf2-ARE pathway (Song et al., 2023). The core biological effect of this activation is enhanced antioxidant capacity, inhibition of inflammation and pyroptosis, thereby conferring protection to multiple systems including the nervous, vascular, and immune systems (Song et al., 2023). Thus, liver-derived fumarate may exert vastly different effects depending on factors such as duration of action, target organ, and concentration. Future studies should further compare mechanistic differences across different models and doses.

#### 3.1.3 α-KG

In liver degenerative diseases such as NASH and cirrhosis, decreased hepatic α-KG levels represent a key change, driven primarily by three mechanisms: firstly, decreased activity of hepatic mitochondrial enzymes (e.g., IDH) directly reduces α-KG synthesis (Li et al., 2024); secondly, elevated oxidative stress in the aging liver compels α-KG to participate more extensively in the glutathione cycle to scavenge reactive oxygen species (ROS), increasing its consumption (Rhoads and Anderson, 2020); thirdly, α-KG is preferentially diverted to the synthesis of amino acids such as proline and glutamate to support collagen production and inflammatory responses (Caffrey et al., 2024). Notably, in contrast to α-KG deficiency in aging tissues, α-KG is aberrantly enriched in some neuroendocrine tumors (Faubert et al., 2020). This suggests tissue-specificity in α-KG metabolism and provides a rationale for targeting this pathway (e.g., developing α-KGDH inhibitors or α-KG prodrugs) for tumor intervention (Zhang B. et al., 2022). Systemic decline in α-KG during aging leads to severe consequences: on one hand, as an essential cofactor for demethylases of the TET and JmjC families, insufficient α-KG causes hypermethylation of DNA and accumulation of repressive histone modifications (e.g., H3K9me3, H3K27me3). This suppresses stem cell functional genes (e.g., Nanog) and regeneration-related pathways (e.g., BMP signaling), ultimately impairing tissue regenerative capacity and accelerating aging (Wang et al., 2020; Ciuffoli et al., 2024). On the other hand, α-KG deficiency inhibits mitochondrial fusion proteins and promotes fission proteins (Cheng et al., 2024), resulting in mitochondrial fragmentation, disruption of cristae structure, and reduced ATP synthesis-outcomes closely associated with conditions such as cognitive decline (e.g., Alzheimer’s disease) (Evans et al., 2021) and sarcopenia (An et al., 2021). Emerging translational studies and early clinical evidence suggest that oral α-ketoglutarate supplementation, following hepatic metabolism and systemic release, may confer protective effects against aging-associated disorders, including muscle decline (Gyanwali et al., 2022). Future studies should further explore the interaction between α-KG and hepatic metabolism, and determine supplementation regimens based on liver function, to provide more precise nutritional intervention strategies for the prevention and treatment of aging-related diseases.

### 3.2 Ketone bodies

Among the three components of ketone bodies, β-hydroxybutyrate (β-HB) is the most central, owing to its high concentration, strong stability, efficient energy supply capacity, and additional metabolic regulatory and protective functions (Aryal et al., 2025). During aging and aging-related pathological contexts, excessive activation of mTORC1 signaling in the liver directly inhibits the transcriptional activity of peroxisome proliferator-activated receptor alpha (PPARα), a key regulator of ketone body production (Sengupta et al., 2010). Reduced PPARα activity leads to decreased expression of the rate-limiting enzyme 3-hydroxy-3-methylglutaryl-CoA synthase 2 (HMGCS2), ultimately suppressing β-HB synthesis (Sengupta et al., 2010). Furthermore, mTOR signaling inhibits autophagy. Concurrently, cellular metabolism shifts from fatty acid β-oxidation to glycolysis, further reducing β-HB production (Singh et al., 2020). In this regard, Moore et al. (Moore et al., 2024) demonstrated that reduced mitochondrial β-HB levels in the liver of NAFLD patients result from the combined effects of multiple factors. The core mechanisms include decreased expression of HMGCS2 (a key enzyme in ketone body synthesis), insufficient substrates due to impaired fatty acid β-oxidation, abnormal MQC, and disrupted PPAR regulatory pathways (Moore et al., 2022; Mooli and Ramakrishnan, 2022). Notably, β-HB levels increase significantly in diabetic ketoacidosis (DKA) or severe stress, while they may remain normal or decrease slightly in states of chronic insulin resistance or long-term hyperglycemia without ketosis (Huang et al., 2024; Min et al., 2023). It is currently proposed that liver-derived β-HB activates the forkhead box O3 (FoxO3a)/metallothionein 2 (MT2) antioxidant pathway by inhibiting histone deacetylases (HDACs). This in turn alleviates oxidative stress, protects mitochondrial function, and ultimately improves myocardial injury and cardiac function (Ji et al., 2022). Additionally, Youm et al. found that elevated circulating β-HB inhibits the NOD-like receptor family pyrin domain containing 3 (NLRP3) inflammasome in a pH-dependent manner and synergizes with free fatty acid receptor 3 (FFAR3) receptor activation. This reduces the secretion of proinflammatory cytokines (IL-1β, IL-18) and pyroptosis, thereby exerting anti-inflammatory effects (Youm et al., 2015). Moreover, increased levels of liver-derived β-HB promote β-hydroxybutyrylation (Kbhb) modification of 3-oxoacid CoA-transferase 1 (OXCT1) and HMGCS2 in a concentration-dependent manner (Fang et al., 2025). This inhibits OXCT1 activity, reduces extrahepatic ketone body utilization via negative feedback, regulates β-HB levels to maintain metabolic homeostasis, and also affects total cellular Kbhb levels (Liśkiewicz et al., 2021). However, no studies have clearly defined the threshold for the beneficial effects of liver-derived β-HB or the impact of the duration of its elevated concentration. These two aspects thus represent key focuses for future research.

### 3.3 Lactate

In the context of NAFLD, HCC, and liver fibrosis, metabolic dysfunction in hepatocytes can form a vicious cycle via lactate-related pathways, exacerbating disease progression and systemic impacts (Cheng et al., 2025). Specifically, during mitochondrial metabolic dysfunction in hepatocytes, hypoxia induces the upregulation of HIF-1α expression (Cheng et al., 2025). As a transcription factor, HIF-1α promotes the expression of lactate dehydrogenase A (LDHA), accelerating the conversion of pyruvate to lactate and preventing pyruvate from entering mitochondria to participate in oxidative phosphorylation (Jin et al., 2025). This leads to lactate accumulation and exacerbation of the Warburg effect, further aggravating hepatocyte injury, inflammation, and fibrosis, and driving the collapse of systemic lipid, glucose, and other metabolic processes (Cheng et al., 2025). During liver fibrosis, mitochondrial metabolic reprogramming in the liver (e.g., enhanced glycolysis) increases lactate production, for example, lactate levels are elevated in CCl_4_-induced liver fibrosis mice (Zhi et al., 2024). This lactate is released from liver fibrotic foci into the circulatory system. As an endogenous ligand for G protein-coupled receptor 81 (GPR81), lactate reaches adipose tissue and binds to GPR81 on the adipocyte surface, reducing lipolysis and thus free fatty acid (FFA) release by inhibiting the cAMP/PKA pathway (Zhi et al., 2024). Although FFA reflux to the liver is reduced, hepatocytes in the fibrotic state still exacerbate hepatic lipid accumulation due to their own metabolic dysfunction (e.g., impaired lipid metabolism) (Zhi et al., 2024). Furthermore, massive accumulation of liver-derived lactate in the circulation causes metabolic acidosis (Rastogi et al., 2023), and can also reduce muscle insulin sensitivity by inhibiting the expression and function of glucose transporter 4 (GLUT4). Mechanistically, lactate activates the AMPK pathway to inhibit the binding capacity of GLUT4 transcription factors, and interferes with the Akt/AS160 pathway to block GLUT4 translocation (Choi et al., 2002).

**FIGURE 4:**
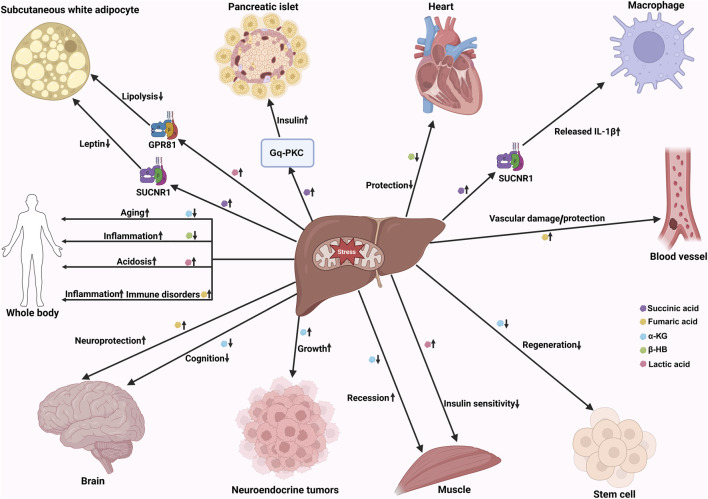
The mechanism of tissue-to-tissue communication mediated by the humoral circulation of liver-derived metabolites and their regulatory effects on multiple organ functions and pathophysiological processes of the whole body. This schematic diagram depicts the interaction between metabolites and specific receptors (such as succinic acid binding sucnr1, lactic acid binding GPR81), as well as the activation of downstream signaling pathways (such as GQ PKC pathway), which ultimately affect the functions of adipose tissue, pancreas, heart, circulating macrophages, blood vessels, brain/nerve and other organs, and participate in key physiological and pathological processes such as inflammation regulation, aging process, insulin sensitivity regulation, neuroprotection and vascular damage repair. Abbreviations: α-ketoglutarate, α-KG; β-hydroxybutyrate, β-HB; succinate receptor 1, SUCNR1; G protein-coupled receptor 81, GPR81; interleukin-1 beta, IL-1β; protein kinase C, PKC.

Overall, as the central organ of metabolism, the liver relies on mitochondria for energy production and their roles in multiple metabolic pathways. Particularly under pathological conditions such as aging and aging-related metabolic diseases, mitochondria maintain metabolic homeostasis by regulating metabolic reprogramming (Kim et al., 2025). Key metabolites, including succinate, fumarate, and α-ketoglutarate, transmit signals inside and outside cells, contributing to inter-organ metabolic crosstalk (Figure 4). However, beyond these well-characterized metabolites, mitochondrial derivatives such as acetyl-CoA, nicotinamide adenine dinucleotide^+^ (NAD^+^), and ATP also play crucial intracellular roles, regulating fatty acid metabolism, DNA repair, epigenetics, and cellular energy status. Future studies should further elucidate how these mitochondrial derivatives precisely regulate metabolic signaling networks, specifically, how they influence cross-organ metabolic regulation via intercellular signal transmission (e.g., exosomes) under different pathological contexts. Investigations into specific mechanisms should focus on the interaction between metabolites and specific receptors, the coordinated regulation of metabolic pathways, and the functional differences of these processes across distinct cell types. Meanwhile, the development of precision therapeutics targeting these metabolites, especially strategies aimed at modulating mitochondrial function and metabolite accumulation to treat aging and aging-related metabolic diseases, is likely to become a promising direction for future research.

## 4 mtROS: from local oxidant to systemic signaling molecule

As the core of cellular energy metabolism, mitochondria produce mtROS, which have long been regarded as the primary source of oxidative stress (Sies et al., 2022; Weinberg and Chandel, 2025). However, recent studies have gradually revealed that mtROS are not only key mediators of intracellular signal regulation, but also systemic signaling molecules capable of spreading across cells and tissues (Weinberg and Chandel, 2025; Casey et al., 2025). Particularly in the context of aging and aging-related diseases, the liver acts as a hub for metabolism and inflammation regulation (Oh et al., 2023). mtROS produced by hepatic mitochondria and damaged mtDNA are no longer confined to intracellular local signals; instead, they are actively released into the circulation, serving as systemic signals that can be “read” by distant tissues (Victorelli et al., 2023; Xiong et al., 2024). The process of converting hepatic metabolic stress into systemic immuno-metabolic reprogramming is a key link driving multi-organ functional remodeling and disease progression (Yang et al., 2025; Giordano et al., 2025).

The traditional view regards mtROS as “leakage products” of the electron transport chain (ETC) (Sies and Jones, 2020; Zhao et al., 2019; Zorov et al., 2014). This perspective stems from early understanding of mtROS production mechanisms: during mitochondrial oxidative phosphorylation, as electrons are transferred along the ETC., a small fraction “leaks” from sites such as Complexes I and III. These leaked electrons combine with oxygen to form superoxide anions (O_2_^−^), which are then converted into other reactive oxygen species (e.g., hydrogen peroxide, H_2_O_2_). This “leakage” is considered a passive, non-specific side reaction that can cause oxidative damage to cells (Weinberg and Chandel, 2025; Zorov et al., 2014). However, recent studies have gradually revealed that mtROS production is not driven solely by ETC., “leakage”. Instead, it can be actively regulated by mechanisms such as reverse electron transport (RET), NAD^+^/nicotinamide adenine dinucleotide (NADH) ratio imbalance, and abnormally elevated mitochondrial membrane potential. Moreover, mtROS exert signaling roles under specific physiological or pathological conditions (Casey et al., 2025; Xu X. et al., 2025). Casey et al. found that during NLRP3 inflammasome activation, macrophages actively generate mtROS via the RET mechanism to regulate IL-1β release—suggesting that mtROS exhibit initiative and targeting in immune regulation (Casey et al., 2025).

Current research tends to define mtROS as regulatable redox signaling molecules, which play critical roles in immunity, inflammatory responses, and aging (Xu X. et al., 2025). During chronic liver injury, excessive ROS production overwhelms the liver’s antioxidant defense system, leading to oxidative stress and cellular damage (Jomova et al., 2023). ROS also directly induce hepatocyte damage and trigger the release of DAMPs, thereby further propagating inflammation and fibrosis (Roehlen et al., 2020). mtROS can be exported extracellularly and activate nuclear factor kappa-light-chain-enhancer of activated B cells (NF-κB) via oxidation; they also synergize with mtDNA to enhance the assembly of NLRP3/NLRC4 inflammasomes, promoting the sustained release of IL-1β/IL-18. This forms an mtROS-inflammation positive feedback loop escalating local oxidative stress into tissue-level inflammation (Patergnani et al., 2021). Furthermore, studies have identified mtROS as an upstream regulatory target of cGAS-STING signaling; mtROS can activate this pathway, thereby triggering hepatic inflammatory responses (Zhang X. et al., 2023). Using a rat model of anti-tuberculosis drug-induced liver injury, Chen et al. found that drug-induced mtROS accumulation causes lysosomal dysfunction and impaired mitophagy. This in turn activates the cGAS-STING pathway, leading to the systemic elevation of inflammatory cytokines such as IFN-β and IL-6. Administration of the mtROS scavenger mitoTEMPO reversed cGAS-STING activation and alleviated liver injury, confirming *in vivo* for the first time that the liver-derived mtROS-cGAS-STING axis functions as a transcellular inflammatory signaling cascade (Chen et al., 2025).

**FIGURE 5:**
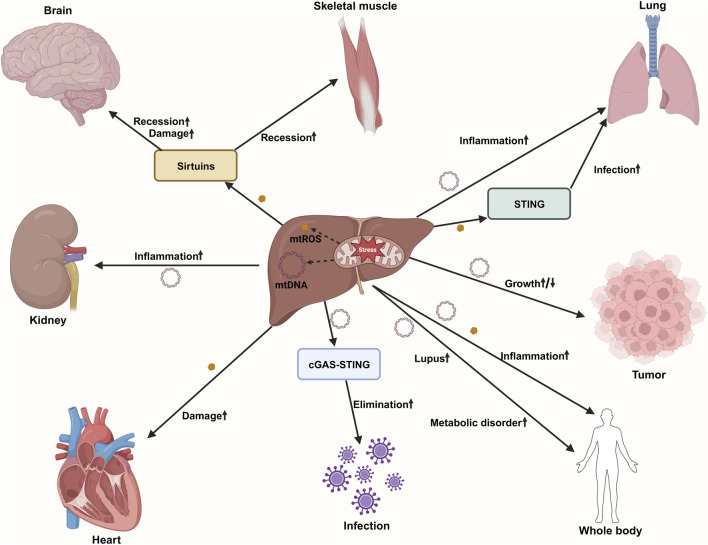
The core mechanism of liver-derived mtROS and mtDNA as key signal molecules affecting multiple organs and physiological and pathological processes through inter-tissue communication. After the release of liver-derived mtROS and mtDNA, it can act on the brain to cause functional decline, induce skeletal muscle injury, lead to lung tissue decline and inflammatory reaction, cause kidney injury, and promote heart infection and related inflammation, etc. Abbreviations: mitochondrial reactive oxygen species, mtROS; mitochondrial DNA, mtDNA; stimulator of interferon genes, STING; cyclic GMP-AMP synthase, cGAS.

At the clinical level, a recent study by Wu et al. demonstrated a positive correlation (r = 0.81) between plasma mtROS levels and Cit-H3, a marker of alveolar neutrophil extracellular traps (NETs), in patients with liver cirrhosis. Platelet-derived HMGB1 activates neutrophils via the receptor for advanced glycation endproducts (RAGE), inducing mtROS bursts and NET formation. This subsequently activates the STING pathway in alveolar macrophages, significantly increasing susceptibility to pulmonary infection (Wu X. et al., 2025). Notably, the systemic role of mtROS is particularly prominent in the context of aging and related pathological contexts. Studies have revealed that elevated mtROS levels in the aging liver are not solely attributed to decreased ETC function, but are closely associated with NAD^+^ depletion and reduced SIRT3 activity—resulting in antioxidant system imbalance and sustained mtROS accumulation (Weinberg and Chandel, 2025). NAD^+^ depletion accelerates the functional decline of the brain and skeletal muscle via the Sirtuins pathway (Gibril et al., 2024; Kolotyeva et al., 2024), reduced SIRT3 activity primarily impairs antioxidant defense, leading to ROS accumulation and subsequent damage to the myocardium and nervous system (Weinberg and Chandel, 2025; Zhong et al., 2022). Together, these evidences support a model in which liver-derived mtROS act as systemic signals that amplify immune dysregulation and organ susceptibility during aging and aging-related diseases, suggesting that targeting mtROS may offer therapeutic opportunities to mitigate systemic aging-associated pathologies (Figure 5).

## 5 The interaction between mtDNA and the immune system

mtDNA exists as a double-stranded circular molecule without histone packaging, adhering closely to the inner mitochondrial membrane. Lacking protection and located in close proximity to the ETC., it is highly susceptible to ROS-induced damage under aging or metabolic stress, leading to oxidative modifications such as 8-oxoguanosine (8-oxoG) (Zhang Z. et al., 2023; He et al., 2022; Yan et al., 2024). If base excision repair enzymes like 8-oxoguanine DNA-glycosylase 1 (OGG1) fail to repair these modifications in a timely manner-due to NAD^+^ depletion or reduced SIRT3 deacetylation activity-oxidized sites rapidly develop into single-strand or double-strand breaks, generating oxidized mtDNA (ox-mtDNA) of 200–650 bp in length (Liu Z. et al., 2022).

In the context of aging and aging-related diseases, sustained release of hepatic ox-mtDNA induces chronic low-grade inflammation in distant tissues such as the lungs and kidneys, driving multi-organ functional decline. Using aged mouse models, Zhong et al. demonstrated that defective mitophagy in hepatocytes leads to the sustained leakage of ox-mtDNA into the circulation. This ox-mtDNA is subsequently taken up by alveolar macrophages and renal tubular epithelial cells, which induces elevated IL-6 and C-X-C chemokine ligand 10 (CXCL10) via the cGAS-STING-NF-κB axis, triggering chronic low-grade inflammation in the lungs and kidneys (Zhong et al., 2022). Aged hepatocytes actively release ox-mtDNA under apoptotic stress, and the circulating ox-mtDNA level correlates positively with IL-1β and TNF-α levels in bronchoalveolar lavage fluid. Upon uptake by distant alveolar macrophages, ox-mtDNA activates the NLRP3 inflammasome, driving persistent alveolar inflammation and impaired gas exchange. This supports the view that ox-mtDNA acts as a “mobile spark” for aging-related multi-organ inflammation (Victorelli et al., 2023). In a systemic aging model, hepatocyte-specific Parkin knockdown leads to ox-mtDNA accumulation and its entry into the bloodstream. Jiménez-Loygorri et al. observed sustained activation of STING and NF-κB in lung and kidney tissues, accompanied by increased collagen deposition and decreased expression of functional proteins. This suggests that ox-mtDNA-mediated chronic low-grade inflammation directly promotes multi-organ functional decline (Jiménez-Loygorri et al., 2024). Thus, ox-mtDNA has a dual identity: it carries both genetic information and oxidative modification. It can cross cell membranes, the circulatory system, and tissue barriers. It converts local hepatic metabolic stress into systemic immuno-metabolic reprogramming, emerging as a druggable target for systemic signaling.

Beyond the aforementioned ox-mtDNA, hepatic mtDNA can also be released into the cytosol under other stress conditions, where it activates immune pathways and subsequently triggers systemic immune responses and systemic inflammatory reactions. These stress conditions primarily include radiation, sepsis-associated liver injury, infection, and ischemia-reperfusion (I/R) injury (Xiong et al., 2024; Giordano et al., 2025; Xu X. et al., 2025; Wang et al., 2025; Wu C. et al., 2025; Jing et al., 2020). For example, I/R injury induces massive hepatocyte necrosis: the cell membrane of necrotic cells ruptures completely, mitochondrial structure disintegrates, and mtDNA is directly released into the extracellular fluid. In apoptotic cells, mitochondrial membrane potential is lost and the outer membrane ruptures locally. mtDNA is first released into the cytosol, then enters the serum via apoptotic bodies or cell membrane leakage (Xiong et al., 2024; Wu C. et al., 2025). In contrast, infectious factors induce hepatic mitochondrial stress or death, leading to mtDNA release via two pathways: firstly, mitochondrial calcium uniporter (MCU)-mediated Ca^2+^ uptake in various cells activates the mitochondrial permeability transition pore (mPTP) (Lai et al., 2023); secondly, reduced mitochondrial membrane potential (ΔΨmt) causes outer mitochondrial membrane (OMM) rupture, releasing mtDNA into the cytosol to activate the cGAS-STING pathway (Lienard et al., 2020). Additionally, dysregulation of any component in MQC prevents the clearance or repair of damaged mitochondria, leading to mitochondrial structural damage and reduced mtDNA stability that ultimately trigger mtDNA release (Wang et al., 2025).

Release of liver-derived mtDNA can contribute to autoimmune diseases, malignancies, and metabolic disorders (Zhang J. et al., 2024; Dai et al., 2024; Liu et al., 2025). Recent studies demonstrate that dysregulation of the cGAS-STING signaling pathway directly promotes the onset and progression of autoimmune diseases such as lupus (Zhang J. et al., 2024). The core mechanism lies in the following: endogenous DNA (particularly oxidized or free mtDNA) released by hepatocytes or mitochondria under stress is recognized by cytosolic DNA sensors (e.g., cGAS), which sustainably induces the production of type I interferons (IFN-I) and proinflammatory cytokines to maintain and amplify pathological autoimmune responses (Kim et al., 2023). Correspondingly, genetic or pharmacological intervention of STING signaling can significantly ameliorate lupus-like phenotypes in mice, underscoring the pathway’s functional role in disease pathogenesis and its potential as a target for regulatory therapies (Alee et al., 2024). Notably, the role of cGAS/STING varies across different experimental models, and differences in intervention timing can trigger opposing immune effects. This observation suggests the pathway follows a “dysregulation-driven pathogenesis” model rather than a simple “overactivation/deficiency” model, with this complexity also providing a mechanistic explanation for clinical heterogeneity (Kumpunya et al., 2022). Further recent studies demonstrate that mtDNA not only acts as a ligand for cGAS to activate the STING pathway, but also exacerbates IFN-I-driven autoimmune inflammation through two additional mechanisms: metabolic-posttranslational modification regulation (e.g., lactylation modification enhances cGAS stability) and the formation of a positive feedback loop with cell death pathways (Zhang J. et al., 2024). Meanwhile, Dai et al. identified a strong association between the onset and progression of NASH and genes related to hepatic mtDNA release through machine learning-based screening (Dai et al., 2024). Thus, these findings demonstrate that targeting mtDNA release, metabolic reprogramming, or direct inhibition of the cGAS/STING pathway may provide novel therapeutic strategies for aging-related disorders (Figure 5).

## 6 Bidirectional organ crosstalk beyond unidirectional liver-derived effects

**FIGURE 6:**
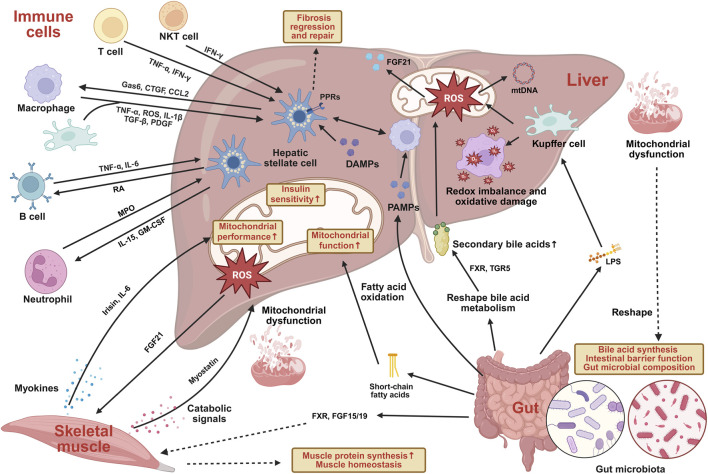
Bidirectional inter-organ crosstalk centered on hepatic mitochondria in aging and aging-related diseases. This schematic depicts bidirectional communication among the gut, skeletal muscle, immune system, and liver, with hepatic acting as an integrative hub. Gut-derived signals, including PAMPs, LPS, bile acid metabolites, and short-chain fatty acids, reach the liver via the portal circulation and modulate mitochondrial redox balance, bile acid metabolism, and inflammatory signaling through FXR and TGR5 pathways. These inputs promote mitochondrial stress, mtROS and mtDNA release, and Kupffer cell activation. Skeletal muscle communicates with the liver through myokines and metabolic cues associated with exercise or sarcopenia, thereby shaping hepatic mitochondrial function and insulin sensitivity. In turn, hepatic mitochondrial stress induces endocrine outputs such as FGF21 that feed back to muscle to regulate protein synthesis and energy homeostasis. Immune cell–hepatic interactions further regulate mitochondrial homeostasis through cytokine- and redox-dependent signaling involving macrophages and hepatic stellate cells. Together, these interconnected axes integrate aging-related metabolic and inflammatory stress into coordinated, bidirectional inter-organ regulation. Abbreviations: farnesoid X receptor, FXR; Takeda G protein-coupled receptor 5, TGR5; mitochondrial reactive oxygen species, mtROS; mitochondrial DNA, mtDNA; fibroblast growth factor 21, FGF21; pathogen-associated molecular patterns, PAMPs; damage-associated molecular patterns, DAMPs; pattern recognition receptors, PPRs; lipopolysaccharide, LPS; natural killer T cells, NKT cells; growth arrest–specific protein 6, Gas6; connective tissue growth factor, CTGF; C-C motif chemokine ligand 2, CCL2; tumor necrosis factor alpha, TNF-α; reactive oxygen species, ROS; interleukin-1 beta, IL-1β; transforming growth factor beta, TGF-β; platelet-derived growth factor, PDGF; interleukin-6, IL-6; retinoic acid, RA; myeloperoxidase, MPO; interleukin-15, IL-15; granulocyte–macrophage colony-stimulating factor, GM-CSF; fibroblast growth factor 15/19, FGF15/19.

Emerging evidence indicates that, beyond unidirectional liver-derived effects, reciprocal signaling operates across the gut-liver, muscle-liver, and immune cell-liver axes (Figure 6). Notably, these three axes do not operate in isolation. Instead, multiple interconnected pathways converge at the hepatic level and collectively shape liver-centered regulation (Ma et al., 2025; Mancin et al., 2023). For example, gut-derived pathogen-associated molecular patterns (PAMPs) can trigger inflammatory activation of hepatic macrophages and drive liver disease progression (Ma et al., 2025). In addition, the gut microbiota can modulate hepatic endocrine output by reshaping bile acid metabolism and the FXR-FGF15/19 signaling axis, thereby translating intestinal metabolic status into systemic signals that influence skeletal muscle protein synthesis and muscle homeostasis (Mancin et al., 2023). In aging and aging-related diseases, the liver acts as a central hub of bidirectional inter-organ communication. Dissecting mitochondria-driven feedback loops linking the gut, skeletal muscle, and immune system will be essential for understanding aging and identifying effective interventions.

### 6.1 Gut-liver axis

In the context of aging and aging-related diseases, the liver-gut axis represents a principal extrinsic regulatory mechanism governing hepatic mitochondrial homeostasis. Mechanistically, approximately 75% of the liver’s blood supply is delivered via the portal vein, which drains blood from the intestine (Bellanti et al., 2023). In addition, hepatic mitochondria and the gut microbiota share multiple conserved proteins that support parallel metabolic pathways (Atlante and Valenti, 2023).

The bidirectional communication within the liver-gut axis primarily involves information and molecular exchange between hepatic mitochondria and the gut microbiota (Clark and Mach, 2017). Alterations in the gut microbiota and its associated metabolites can promote excessive mtROS production, leading to redox imbalance and oxidative damage, which are key pathogenic drivers of steatosis progression and the development of steatohepatitis in metabolic dysfunction-associated NAFLD (Loomba et al., 2017). Evidence indicates that fecal microbiota transplantation from donors with hyperglycemia and elevated circulating pro-inflammatory cytokines can induce hepatic macrovesicular steatosis and increase the expression of genes involved in *de novo* lipogenesis (Le Roy et al., 2013). Mechanistically, microbiota-derived lipopolysaccharide can chronically translocate to the liver and activate Kupffer cells, thereby amplifying hepatic mitochondrial oxidative stress and promoting the release of mitochondrial danger signals, such as mtROS and mtDNA (Zhang Q. et al., 2022). In parallel, gut microbiota-dependent bile acid remodeling generates secondary bile acids that signal through FXR and TGR5, thereby linking microbial composition to hepatic mitochondrial stress responses and mitochondria-associated endocrine outputs, including FGF21 (Biagioli and Fiorucci, 2021). Moreover, microbial metabolites such as short-chain fatty acids, particularly butyrate, have been shown to support hepatic mitochondrial function by enhancing fatty acid oxidation (Mollica et al., 2017). Consistent with the mechanisms discussed above, hepatic mitochondrial dysfunction can also influence intestinal homeostasis. For example, impaired mitochondrial regulation of bile acid synthesis (Kakiyama et al., 2023) and increased release of mitochondrial danger signals, such as mtDNA (Zhang Q. et al., 2022), reshape gut microbial composition and intestinal barrier function (LeFort et al., 2024), thereby reinforcing gut-liver communication in aging-related liver diseases. Collectively, the liver-gut axis emerges as a bidirectional pathway through which aging-related disturbances are integrated at the level of hepatic mitochondria, shaping disease susceptibility and progression in chronic liver disorders.

### 6.2 Muscle-liver axis

Skeletal muscle functions as an endocrine organ and influences hepatic metabolism through circulating myokines, extending mitochondrial regulation beyond liver-intrinsic pathways (Dumond et al., 2023). Changes in muscle state, including exercise adaptation and muscle reduction, are conveyed to the liver and shape hepatic mitochondrial function and metabolic flexibility. Exercise-associated myokines such as irisin (Ciałowicz et al., 2025) and IL-6 (Mao et al., 2025) are linked to improved hepatic insulin sensitivity and mitochondrial performance, whereas catabolic signals released during sarcopenia, including myostatin, are associated with impaired hepatosteatosis and mitochondrial dysfunction (Joo and Kim, 2023). Importantly, muscle-liver communication operates within a reciprocal framework. As mentioned earlier, as hepatic mitochondrial stress responses induce mitochondria-linked liver-derived factors, such as FGF21 (Keipert and Ost, 2021), which can in turn modulate skeletal muscle metabolism and function. Together, the muscle-liver axis represents a bidirectional system coordinating peripheral energy demand with hepatic mitochondrial adaptation.

### 6.3 Immune cell-hepatic crosstalk

Immune cell-hepatic crosstalk represents another critical extrinsic layer shaping hepatic mitochondrial homeostasis in aging-related liver diseases. Hepatic stellate cell (HSC)-immune cell crosstalk is most clearly exemplified by interactions with macrophages (Matsuda and Seki, 2020). Following liver injury, bidirectional signaling between HSCs and macrophages coordinates activation of both cell populations (Carter and Friedman, 2022). In this reciprocal relationship, HSCs can further potentiate the fibrogenic activity of macrophages. This finely tuned interplay has recently been conceptualized as a paracrine, two-cell circuit that stabilizes their mutual states and largely predicts their behaviors in liver physiological and pathological conditions (Zhou et al., 2018; Adler et al., 2020). Interactions between HSC and other immune populations are less comprehensively characterized, yet remain important. In experimental fibrosis models, HSC activation is reduced in immunodeficient (SCID) mice and is restored by adoptive transfer of lymphocytes, particularly CD8^+^ T cells (Safadi et al., 2004). Similarly, in mice subjected to CCl4-induced liver injury, B cells and HSCs establish a pro-fibrogenic network. Retinoic acid signaling from HSCs supports B cell survival and activation, whereas B cells, in turn, secrete inflammatory cytokines (Thapa et al., 2015). In other settings, immune cells can restrain HSC fibrogenic responses. Neutrophils, through signaling to macrophages, promote the transition from disease progression to resolution, a phase in which liver injury has ceased and inflammatory programs shift globally toward repair, including fibrosis regression (Calvente et al., 2019). Likewise, interferon-γ (IFNγ), produced by multiple immune cell types including NK and T cells, exerts direct anti-fibrogenic effects on HSCs (Zhang et al., 2017; Araujo et al., 2018; Aguilar-Valenzuela et al., 2014).

Consistent with these broader principles, interactions between HSCs and hepatic immune cells are key regulators of fibrosis in NASH. HSCs cooperate with innate immune cell populations to initiate hepatic inflammation during the transition from simple steatosis to steatohepatitis. They also undergo fibrogenic activation in response to inflammatory cues delivered by both innate and adaptive immune cells and subsequently signal back to these subsets, thereby amplifying their activation (Carter and Friedman, 2022; Loomba et al., 2021). Collectively, these findings highlight immune cell-HSC crosstalk as a dynamic regulatory network that integrates inflammatory signals to shape hepatic fibrogenesis and mitochondrial homeostasis across liver health and disease.

## 7 Therapeutic potentials of targeting hepatic mitochondrial-related factors

We summarize how hepatic mitochondrial-related factors act as a “central hub” influencing systemic organ function in aging and aging-related metabolic diseases, with an emphasis on their translational potential (Table 1). Currently, the clinical translation of mitochondrial-related factors can be broadly categorized into two strategies: one that directly targets the mitochondrial-related factors themselves, and another that acts on their target organ receptors. On one hand, drugs can modulate receptor sensitivity to mitochondrial-related factors, thereby enhancing their beneficial effects on organ systems affected by aging and related pathological contexts. On the other hand, interventions may directly inhibit these factors or develop novel agents that “mimic or replace” their function. In contrast, direct pharmacological modulation of hepatic mitochondrial function remains underdeveloped. Although liver-derived mitochondrial signaling has attracted growing interest, most available mitochondrial modulators lack hepatocyte selectivity and act broadly across tissues. This limits the ability to ascribe systemic metabolic or inflammatory effects specifically to hepatic mitochondrial perturbation. Consequently, direct evidence linking targeted manipulation of liver mitochondria to coordinated responses in distant organs is still scarce. These limitations largely reflect the absence of liver-selective delivery approaches, the strong conservation of mitochondrial pathways across tissues, and incomplete understanding of when and under which conditions hepatic mitochondrial signals exert adaptive versus maladaptive effects. Rather than broadly targeting mitochondrial machinery, current translational progress has therefore concentrated on downstream, more accessible signaling nodes, particularly UPRmt-induced circulating factors and their receptors. This shift has enabled the development of pharmacological strategies that act on defined signaling pathways, which are discussed in the following section.

**TABLE 1:** Summary of liver-derived mitochondrial signals and their multi-organ effects in aging and aging-related metabolic diseases.

Factor	Stress factor	Expression	Effect	Ref.
FGF21	Amino acid deficiency	↑	Promote fatty acid oxidation and ketone formation in liver	Szczepańska and Gietka-Czernel (2022)
			Promote hypothalamic energy balance and neuroendocrine secretion	Szczepańska and Gietka-Czernel (2022)
			Increase systemic insulin sensitivity	Fisher and Maratos-Flier (2016); De Sousa-Coelho et al. (2023)
	Enhanced fatty acid oxidation		Increase glucose uptake and lipolysis in adipose tissue	Kaur et al. (2022); Fisher and Maratos-Flier (2016); De Sousa-Coelho et al. (2023); Szczepańska and Gietka-Czernel (2022); Shin et al. (2023)
			Myocardial protection	Kaur et al. (2022); Fisher and Maratos-Flier (2016)
			Neuroprotective	Fang et al. (2020)
			Carcinogenesis	Hu et al. (2024)
GDF15	Cellular damage, inflammation, hypoxia, and metabolic disorders cause liver UPRmt activation	↑	Increase systemic insulin sensitivity and energy expenditure	Jena et al. (2023); Salminen (2024)
	Chemotherapy drugs activate the IRE1α-XBP1 signaling pathway		Promote lipolysis of adipose tissue	Reyes et al. (2024); Du et al. (2025)
			Promote the development of malignant tumors	Ling et al. (2023)
			Promote muscle atrophy	Salminen (2024)
			Promote severe infection	Reyes et al. (2024)
Succinic acid	Inhibition of SDH activity by hypoxic microenvironment or Warburg effect	↑	Promote the release of IL-1β from macrophages in circulation	Rawal et al. (2024)
	IDH1/2 mutation indirectly inhibits mitochondrial SDH activity		Inhibit the expression of leptin in adipose tissue	Villanueva-Carmona et al. (2023)
			Promote insulin secretion of pancreatic islets	Sabadell-Basallote et al. (2024)
Fumaric acid	NASH	↑	Remodeling the metabolic pattern of tumor associated macrophages	Lu et al. (2025)
	Toxic exposure		Promote systemic inflammatory response, immune imbalance and metabolic disorder	Hoyle et al. (2022)
			Cause vascular injury such as aortic dissection	Lian et al. (2019)
			Protect nervous, vascular, immune and other systems	Song et al. (2023)
α-KG	NASH	↓	Damage tissue regeneration and accelerate aging	Wang et al. (2020); Ciuffoli et al. (2024)
	Liver cirrhosis		Promote cognitive decline	Evans et al. (2021)
	As a raw material for synthesizing proline and glutamic acid		Promote muscle loss	Ciuffoli et al. (2024)
β-HB	Aging	↓	Relieve systemic inflammatory response	Youm et al. (2015)
	NAFLD		Improve myocardial injury and cardiac function	Shin et al. (2023)
	Chronic insulin resistance or long-term hyperglycemia without ketosis			
Lactic acid	NAFLD	↑	Inhibition of lipolysis of adipose tissue	Zhi et al. (2024)
	Liver cancer			
	Liver fibrosis			
			Cause systemic metabolic acidosis	Rastogi et al. (2023)
			Decrease muscle insulin sensitivity	Choi et al. (2002)
mtROS	ETC., “leakage”	↑	Increase the risk of pulmonary infection	Wu et al. (2025a)
	RET		Promote brain function decline	Kolotyeva et al. (2024)
	Imbalance of NAD^+^/NADH ratio		Promote the decline of skeletal muscle function	Gibril et al. (2024)
	Abnormal increase in mitochondrial membrane potential		Cause myocardial injury	Weinberg and Chandel (2025)
	Aging		Promote systemic inflammatory response	Weinberg and Chandel (2025); Zhong et al. (2022)
	Liver cirrhosis		Promote nerve injury	Weinberg and Chandel (2025)
mtDNA	Aging	↑	Promote renal inflammation	Zhong et al. (2022)
	Radiation		Accelerate pathogen clearance	Dai et al. (2024)
	Sepsis related liver injury		Cause autoimmune disease	Zhang et al. (2024c)
	Infection		Cause systemic metabolic disorder	Dai et al. (2024)
	Ischemia-reperfusion injury		Cause lung inflammation	Zhong et al. (2022)
			Inhibit/promote tumor growth	Dai et al. (2024); Liu et al. (2025)

Abbreviations: fibroblast growth factor 21; FGF21, growth differentiation factor 15; GDF15, α-ketoglutarate; α-KG, β-hydroxybutyrate; β-HB, mitochondrial reactive oxygen species; mtROS, mitochondrial DNA; mtDNA, mitochondrial unfolded protein response; UPRmt, inositol-requiring enzyme 1 alpha; IRE1 α, X-box binding protein 1; XBP1, succinate dehydrogenase; SDH, isocitrate dehydrogenase 1 and 2; IDH1/2, nonalcoholic steatohepatitis; NASH, nonalcoholic fatty liver disease; NAFLD, electron transport chain; ETC, reverse electron transport; RET, nicotinamide adenine dinucleotide+; NAD+, nicotinamide adenine dinucleotide; NADH, interleukin-1; beta; IL-1β.

### 7.1 Targeting UPRmt-driven cross-tissue signaling

#### 7.1.1 Targeting FGF21

FGF21 and GDF15, although no drugs targeting them have yet been approved for clinical use, have been the focus of extensive translational research. Studies have shown that GLP-1 receptor agonists, such as semaglutide and liraglutide, can acutely stimulate hepatic FGF21 secretion. More importantly, they upregulate KLB and FGFR expression in epididymal adipose tissue, thereby enhancing systemic FGF21 sensitivity under chronic conditions (Feng et al., 2023; Le et al., 2023). Beyond pharmacological interventions, Liu et al. reported in multiple animal and clinical follow-up studies that bariatric surgery or substantial weight loss can restore FGF21 signaling sensitivity, potentially through upregulation of receptors or downstream responses, and they observed a functional “recovery” of FGF21 signaling (Liu et al., 2019). Similarly, exercise has been shown to promote KLB and FGFR expression in cardiac and adipose tissues, enhancing systemic responsiveness to FGF21 (Geng et al., 2019). Regarding the pro-tumorigenic effects of FGF21, Hu et al. found that anti-FGF21 neutralizing antibodies can directly neutralize circulating or tumor-derived FGF21, restoring CD8^+^ T cell function and alleviating immunosuppression induced by altered lipid or cholesterol metabolism (Hu et al., 2024). However, this work remains at the experimental stage. Currently, research on β-Klotho antagonistic peptides has reached the preclinical stage (Pan et al., 2020), these peptides act by blocking the interaction between β-Klotho-FGFR and FGF21, thereby inhibiting FGF21 signaling and exerting anti-cancer effects.

#### 7.1.2 Targeting GDF15

Modulating hepatic release of GDF15 into the circulation has emerged as a novel therapeutic target. For example, metformin and halofuginone can markedly increase circulating GDF15 via the hepatic eIF2α-ATF4 pathway (Day et al., 2019; Xu S. et al., 2025). This hepatically derived GDF15 plays a key role in the weight-reducing effects of metformin and halofuginone (Xu S. et al., 2025; Zhang S-Y. et al., 2023). Likewise, in certain malignancies such as cancer, antibodies targeting GDF15 have been developed. Currently, AZD8853 and visugromab have entered early clinical trials. They demonstrate good tolerability in patients with advanced solid tumors, but their efficacy and durability of inhibition require further validation (Carneiro et al., 2025; Melero et al., 2025).

### 7.2 Targeting TCA cycle intermediates

#### 7.2.1 Targeting succinate

Haffke et al. and Taghavi et al. have explored strategies to treat NASH and other inflammatory diseases either by antagonizing SUCNR1 or by reducing succinate production or release (Taghavi et al., 2022; Haffke et al., 2019). Haffke et al. demonstrated the feasibility of NF-56-EJ40, a high-affinity and human-selective SUCNR1 antagonist, through high-resolution structural analysis of the humanized receptor–antagonist complex and functional assays. This work provides a “druggable” structural framework for small molecules and biologics targeting SUCNR1 (Haffke et al., 2019). Besides, Taghavi et al. administered exogenous dimethyl malonate (DMM) in animal models, which slowed plasma succinate accumulation, mitigated organ dysfunction, and improved short-term physiological parameters. These findings suggest that metabolic interventions can reduce circulating succinate and attenuate its pro-inflammatory effects (Taghavi et al., 2022).

#### 7.2.2 Targeting fumaric acid

Research on therapeutic targets for fumaric acid remains limited, with most studies focusing on ameliorating fumaric acid-induced NASH and neurological disorders. Evidence shows that lifestyle interventions, such as moderate exercise, can induce hepatic kruppel-like factor 10 (Klf10) expression via the cAMP/PKA/CREB pathway, thereby restoring or even enhancing fumarate hydratase (FH) activity, reducing hepatic fumarate accumulation, and partially reversing NASH (Luo et al., 2024). Bresciani et al. demonstrated that dimethyl fumarate (DMF) effectively alleviates neurological disorders caused by fumarate-associated oxidative damage (Bresciani et al., 2023).The mechanism involves activation of the KEAP1-NRF2 antioxidant pathway, which suppresses inflammatory responses. However, it is important to note that DMF may exert opposite effects in different tissues depending on the dose.

#### 7.2.3 Targeting α-KG

As mentioned above, oral supplementation of α-KG to elevate systemic α-KG levels can correct aging-associated metabolic and epigenetic alterations. A representative translational effort in humans was a randomized controlled trial in middle-aged individuals designed and initiated by Sandalova et al. (2023). The study revealed that participants receiving oral α-KG showed significant improvements in DNA methylation age, systemic inflammation, grip strength, metabolic parameters, and other aging-related measures compared with the placebo group, indicating that α-KG supplementation has entered clinical validation. In addition, studies have shown that α-KG supplementation can modulate tumor metabolism. For example, in animal and cellular models of DLBCL (diffuse large B-cell lymphoma), increased α-KG inhibited tumor growth and altered metabolic profiles (Cai et al., 2023), suggesting that direct α-KG supplementation may have anti-tumor potential in certain cancers and could represent a promising future strategy for cancer therapy.

### 7.3 Targeting β-HB

In most inflammation and stress models, moderate elevation of β-HB improves mitochondrial function. Recent early-phase human and animal studies have shown that administration of exogenous ketone esters can robustly elevate circulating β-HB in the short term, exerting NLRP3 inhibitory effects and metabolic protective signaling (Mohammed et al., 2024). Additionally, short-term fasting and ketogenic interventions can increase circulating β-HB, suppress NLRP3 activation, and confer protection in multi-organ inflammation models (Neudorf and Little, 2024). Therefore, β-HB can be considered a controllable pharmacological or nutritional intervention for studies of anti-inflammatory and metabolic protection. However, it is important to note that the potential pro-pathogenic effects of β-HB, depending on dosage or specific tumor contexts, cannot be entirely excluded. Precise control of circulating β-HB levels remains a key challenge for future applications.

### 7.4 Targeting lactate

In recent years, the clinical feasibility of targeting lactate and its potential in combination immunotherapy have been extensively validated. The main pharmacological strategies center on limiting lactate production and inhibiting its transmembrane transport. Verma et al. used the LDH inhibitor GNE-140 in mouse tumor models and *in vitro* experiments to reduce lactate production by tumor cells and increase glucose availability in the tumor microenvironment. They observed enhanced T cell metabolism and effector function, resulting in significantly improved responsiveness to anti–PD-1/PD-L1 therapy (Verma et al., 2024). Similarly, in glioblastoma and transplant models, Khan et al. demonstrated that LDH inhibition suppresses macrophage chemotaxis and slows tumor progression (Khan et al., 2024). In this regard, Halford et al. conducted more advanced studies on the MCT1 inhibitor AZD3965, which has entered early-phase dose-escalation and expansion cohort trials in humans. AZD3965 blocks lactate transmembrane transport, disrupting lactate efflux and exchange, thereby altering the acidic tumor microenvironment and inducing metabolic vulnerability. The drug has demonstrated safety and tolerability in humans while achieving mechanistic inhibition (Halford et al., 2023). Despite encouraging early results, the clinical efficacy of lactate-targeted therapies remains incompletely defined. Metabolic plasticity, compensatory lactate pathways, and unresolved questions regarding efficacy and patient selection remain key obstacles to clinical translation (Dong et al., 2025). Future research targeting lactate in liver metabolic pathways may prioritize combinatorial mechanisms and patient-specific therapeutic approaches.

### 7.5 Targeting mtROS

Targeted mtROS scavenging has emerged as a cutting-edge research direction for addressing hepatic mitochondrial oxidative stress injury, with core mechanisms categorized into mtROS clearance and inhibition of mtROS release. For instance, Mito-TEMPO can precisely restore hepatic mitochondrial function and activate antioxidant pathways to clear mtROS. This not only promotes liver recovery, but also inhibits systemic inflammation and pyroptosis by regulating tissue crosstalk mediated by cytokines and DAMPs (Wang PF. et al., 2022). In contrast, elamipretide reduces mtROS release by stabilizing mitochondrial membranes, thereby alleviating liver injury and suppressing excessive systemic inflammatory responses (Sabbah et al., 2025). However, progress in the clinical translation of such drugs remains hindered by factors including insufficient evidence of positive efficacy from human trials, challenges in achieving precise drug dosage control, and inter-individual disease heterogeneity. Future research should focus on biomarker-guided personalized combination therapies and precision medicine approaches, to ensure the sustainability and broad applicability of these strategies in clinical practice.

### 7.6 Targeting mtDNA

In recent years, research on mtDNA aberrant release–driven autoimmune and metabolic diseases has made significant breakthroughs, particularly in the development of drugs targeting the cGAS-STING signaling axis (Maekawa et al., 2023). The core strategies focus on three types of interventions: blocking mtDNA recognition, inhibiting downstream inflammatory signaling, and modulating amplification loops of effector cytokines (Yu et al., 2024). The cGAS inhibitor VENT-03, developed by Ventus Therapeutics, is the first representative molecule to enter clinical trials. VENT-03 directly blocks cGAS DNA binding and cGAMP synthesis activity, effectively interrupting the mtDNA-induced IFN-I amplification loop. In a 2024 phase I trial (n = 72), VENT-03 demonstrated good tolerability and robust pharmacodynamic signals, achieving complete *in vivo* cGAS target inhibition and laying the foundation for phase II studies in mtDNA-driven diseases such as systemic lupus erythematosus (SLE) (Pike et al., 2024). This represents the first feasibility study in humans to validate restoration of immune homeostasis via direct intervention at the mtDNA recognition step. Meanwhile, AstraZeneca’s anti-IFNAR1 monoclonal antibody anifrolumab has successfully validated a downstream inhibition strategy by blocking IFN-I receptor signaling. In the TULIP-2 study (n = 362), anifrolumab significantly improved disease activity in patients with moderate-to-severe SLE and reduced corticosteroid dependence, clinically confirming the therapeutic value of inhibiting terminal signaling of the mtDNA-cGAS-STING-IFN axis (Morand et al., 2020). In addition, the NLRP3 inflammasome inhibitor dapansutrile blocks mtDNA-induced inflammasome assembly and IL-1β release, demonstrating good safety and biological activity in phase I-II clinical trials for chronic heart failure and gouty arthritis (Wohlford et al., 2020). These results establish NLRP3 as a key target within the mtDNA-inflammation coupling network.

## 8 Bioenergetic interventions for aging and aging-related diseases

Bioenergetic interventions such as caloric restriction and regular exercise has been shown to delay aging and aging-related diseases (Cutuli et al., 2023; Millan-Domingo et al., 2024). In parallel, a growing number of clinical trials targeting aging are underway, providing increasing evidence for the safety and efficacy of these interventions (Wang K. et al., 2022).

### 8.1 Caloric restriction mimetics

Caloric restriction (CR) is one of the most robust non-pharmacological interventions known to extend lifespan and improve metabolic health, and its beneficial effects are closely linked to adaptive remodeling of hepatic mitochondrial function (Yeh et al., 2026).

A growing body of evidence supports the existence of pharmacological caloric restriction mimetics (CRMs), defined as compounds that reproduce key molecular and physiological features of caloric restriction without sustained reductions in energy intake (Hofer et al., 2021). To date, multiple pharmacological agents capable of mimicking caloric restriction have been identified, including metformin, rapamycin, resveratrol, spermidine, and acarbose (Ingram and Roth, 2015; Martel et al., 2021; Madeo et al., 2019). Metformin directly inhibits mitochondrial electron transport chain complex I and subsequently induces protective autophagy in the liver through the AMPK–mTORC1 signaling axis, effects that extend lifespan in animal models and are associated with a reduced risk of metabolic syndrome, cardiovascular disease, and cancer in human populations (Madeo et al., 2019). Recent mechanistic work has refined this view by demonstrating that rapamycin-induced autophagy is not solely a consequence of mTORC1 inhibition but is amplified through metabolic rewiring of polyamine synthesis. Specifically, caloric restriction and rapamycin both elevate intracellular spermidine levels, which promote EIF5A hypusination and enhance translation of the master lysosomal and autophagy regulator TFEB (Hofer et al., 2024a). Spermidine induces autophagy through activation of the polyamine-eIF5A hypusination pathway and is required for caloric restriction adaptation, thereby preserving mitochondrial metabolic function and supporting lifespan and healthspan extension across species (Hofer et al., 2024b). Together, existing studies position caloric restriction mimetics as powerful modulators of hepatic mitochondrial programs. However, incomplete understanding of dose, time, and context-dependent effects underscores the need for future work to disentangle adaptive versus maladaptive mitochondrial signaling and to translate these mechanisms into safe and durable interventions.

### 8.2 Exercise mimetics

Exercise is a potent physiological stimulus that remodels hepatic mitochondrial function and contributes to systemic metabolic adaptation (Esteves and Stanford, 2024). As discussed above, moderate exercise reduces hepatic fumarate accumulation and may even reverse NASH (Luo et al., 2024). Evidence indicates that acute exercise rapidly activates hepatic mitophagic flux through ubiquitin- and receptor-dependent pathways, promoting the selective removal of dysfunctional mitochondria. This adaptive remodeling preserves mitochondrial quality and supports post-exercise systemic metabolic flexibility and glucose homeostasis (Amar et al., 2024; McCoin et al., 2022). This process preserves hepatic MQC and supports post-exercise systemic metabolic remodeling and glucose homeostasis. In addition, exercise mimetics such as GW501516 and SLU-PP-332 improve overall metabolic phenotypes by modulating mitochondria-associated factors. Specifically, GW501516 upregulates GDF15 via the hepatic PPARβ/δ-AMPK-p53 axis, thereby promoting systemic fatty acid oxidation and improving glucose tolerance (Aguilar-Recarte et al., 2021). By contrast, SLU-PP-332 enhances whole-body energy expenditure and fatty acid oxidation through ERR-regulated hepatic mitochondrial fatty acid oxidation and respiratory programs, leading to improved insulin sensitivity and reduced lipid accumulation (Billon et al., 2024). These observations support the view that hepatic mitochondrial adaptations serve as a key hub through which exercise and exercise mimetics drive systemic metabolic remodeling.

## 9 Mitokine-based biomarker development

Mitokine-based biomarkers provide a practical link between hepatic mitochondrial stress and clinically measurable signals. At present, FGF21 and GDF15 testing is not routinely performed in hospital laboratories (Tan et al., 2023; Nilsen et al., 2024). However, accumulating evidence indicates that both FGF21 and GDF15 are upregulated during aging and in multiple aging-related diseases, supporting their potential value as diagnostic and prognostic biomarkers in these settings (Tan et al., 2023). FGF21 and GDF15 have been widely studied as biomarkers for metabolic disorders, including obesity, metabolic syndrome, insulin resistance, and type 2 diabetes (Yang et al., 2023; Chuang et al., 2025). Beyond metabolic disease, FGF21 shows promise for assessing disease severity and predicting long-term outcomes. In chronic kidney disease (CKD), elevated circulating FGF21 levels are consistently associated with more advanced disease stages and poorer clinical prognosis (Yong et al., 2023). Similarly, in sepsis and other critical illnesses, higher FGF21 levels tend to correlate with worse disease severity and adverse prognosis (Li et al., 2021). Moreover, one study suggests that FGF21 may outperform other adipokines and could be proposed as an alternative to the glucose tolerance test (Woo et al., 2017). GDF15 also displays strong biomarker potential across several disease contexts. In CKD, elevated GDF15 levels are associated with accelerated renal function decline and an increased risk of disease progression (Delrue et al., 2023). In addition, tumor burden and inflammatory stress can increase circulating GDF15 levels, and GDF15 has been discussed as a key component of cachexia-associated pathways and a potential therapeutic target (Hüllwegen et al., 2025). With advancing age, circulating levels of GDF15 generally show an increasing trend (Conte et al., 2022). Besides, in elderly cohorts, higher GDF15 levels are frequently associated with greater frailty, poorer physical performance, and malnutrition (Nielsen et al., 2024; Merchant et al., 2023). Collectively, future research on mitokine-based biomarker development should prioritize standardized measurement, longitudinal validation across disease stages, and mechanistic clarification of how tissue-specific mitochondrial stress shapes circulating FGF21 and GDF15 signals, thereby enabling their reliable translation into clinically actionable tools for aging and aging-related diseases.

## 10 PGC1α-targeted therapies

PPARγ co-activator PGC-1α is a key controller of mitochondrial biogenesis and oxidative metabolism, and targeting this axis can reshape hepatic mitochondrial programs with systemic consequences (Abu et al., 2023; Rius-Pérez et al., 2020; de Oliveira Bristot et al., 2019). In metabolic stress settings, exercise training can restore reduced hepatic PGC-1α expression, consistent with a re-engagement of mitochondrial quality-related programs in the liver (Dethlefsen et al., 2018). At the same time, the liver context matters, hepatic PGC-1α can also drive gluconeogenesis, and excessive or unbalanced activation may therefore worsen glucose handling. Supporting this, osteocalcin stimulation increases PGC-1α expression in hepatocytes and liver explants, and in the absence of GLP-1 receptor signaling this response is linked to enhanced hepatic glucose production and glucose intolerance (Mizokami et al., 2020). Pharmacologically, PPAR agonists represent an indirect route to elevate PGC-1α-linked mitochondrial programs, as exemplified by bezafibrate, which activates PPAR signaling and increases PGC-1α expression to promote mitochondrial biogenesis in cellular models (Augustyniak et al., 2019). In parallel, small molecules that enhance PGC-1α activity, such as ZLN005, can improve mitochondrial homeostasis in disease models in a manner that depends at least in part on PGC-1α (Zhu et al., 2022). Together, these data support PGC-1α as a tractable lever to tune liver mitochondrial function, while emphasizing that therapeutic benefit will depend on achieving a balanced mitochondrial program without exacerbating hepatic glucose overproduction.

## 11 Conclusions and future perspectives

The concept that hepatic mitochondria serve not merely as metabolic engines but as command centers orchestrating inter-organ communication redefines our understanding of organismal aging and aging-related diseases. By decoding how mitochondrial stress is translated into systemic endocrine, metabolic, and immune signals, we are beginning to appreciate the liver as both a metabolic sensor and a molecular broadcaster of aging trajectories. This framework positions hepatic mitochondria at the heart of a cross-tissue dialogue that integrates nutrient sensing, inflammatory tone, and energy distribution across the body (Table 1). Specifically, hepatic mitochondria convert local metabolic stress into systemic immuno-metabolic signals by regulating mitochondrial factors and metabolites. These findings not only uncover the shared pathological mechanisms underlying diseases such as NAFLD, Parkinson’s disease, osteoporosis and aortic dissection, but also propose an innovative paradigm that positions hepatic mitochondria as a hub for anti-aging interventions (Figure 2). Following this paradigm, a growing number of therapeutic interventions targeting hepatic mitochondrial-related factors have emerged (Table 2). Several successful agents, such as semaglutide, liraglutide, and metformin, have already been approved for clinical use, alongside preventive strategies including exercise and bariatric surgery (Feng et al., 2023; Le et al., 2023; Day et al., 2019). These interventions effectively mitigate adverse outcomes associated with dysregulated mitochondrial signaling, not only extending lifespan but, more importantly, substantially improving patients’ quality of life. Numerous other candidates, such as visugromab and NF-56-EJ40, remain at the preclinical or animal study stage. Although their clinical translation is currently hindered by uncertainties in efficacy stability, optimal dosing, and temporal thresholds, as well as by challenges related to organ-specific delivery and complex signaling dynamics, these agents nonetheless demonstrate remarkable translational potential.

**TABLE 2:** Summary of therapeutic strategies targeting liver-derived mitochondrial factors and their translational relevance.

Interfere	Model	Target	Mechanism	Translational stage	Ref.
Semaglutide	High-fat diet-induced diabetic and obese mouse models	FGF21	Activation of GLP-1R stimulates hepatic FGF21 expression and upregulates FGFR and KLB, transiently restoring FGF21 sensitivity suppressed by high-fat challenge and thereby improving lipid and glucose metabolism	Clinical approved	Feng et al. (2023)
Liraglutide	Human and rodent (mouse and rat) models of obesity and metabolic syndrome		By activating both central and peripheral GLP-1Rs, GLP-1 analogues promote hepatic FGF21 production and upregulate FGFR and KLB in hepatic and adipose tissues, enhancing FGF21 responsiveness in target organs and improving insulin sensitivity and body weight	Clinical approved	Le et al. (2023)
Anti-FGF21 neutralizing antibody	Tumor-bearing mouse models (syngeneic and xenograft)		Direct neutralization of circulating or tumor-derived FGF21 blocks its signaling through KLB/FGFR, restores CD8^+^ T-cell metabolism and cytotoxicity by reversing FGF21-induced cholesterol metabolic reprogramming, and suppresses tumor progression	Preclinical	Hu et al. (2024)
β-Klotho antagonist peptide	Mouse models with pharmacologic/genetic FGF21 activation; *in vitro* cell systems		Competitive peptides that disrupt the interaction between FGF21 and KLB inhibit receptor complex activation, thereby antagonizing FGF21 signaling and exhibiting antitumor potential in specific cancer models	Preclinical	Pan et al. (2020)
Metformin	Murine and human metabolic models	GDF15	Activation of the eIF2α–ATF4/CHOP pathway upregulates hepatic and renal GDF15 expression, mediating appetite suppression and metabolic improvement	Clinical approved	Day et al. (2019)
Halofuginone	Diet-induced obese mouse models		Induction of hepatic or systemic stress pathways elevates GDF15 (and potentially FGF21) expression, leading to appetite suppression and enhanced energy expenditure	Preclinical	Xu et al. (2025b)
AZD8853	Advanced/metastatic solid tumor patients		Anti-GDF15 monoclonal antibodies neutralize tumor-derived or circulating GDF15 to relieve GDF15-mediated immunosuppression and restore antitumor immunity	Clinical phase I–II	Carneiro et al. (2025)
Visugromab	Clinical cohorts of advanced solid tumors and neoadjuvant bladder cancer		Anti-GDF15 monoclonal antibodies neutralize tumor-derived GDF15 and are evaluated in combination with immunotherapy or chemotherapy for their potential to potentiate immune responses	Clinical phase I–II	Melero et al. (2025)
NF-56-EJ40	*In vitro* receptor assays; mouse inflammatory models	succinate	Selective high-affinity antagonists of SUCNR1 block succinate-SUCNR1-mediated inflammatory signaling	Preclinical	Haffke et al. (2019)
DMM	Porcine and murine hemorrhagic/ischemia–reperfusion injury models		Modulating or redirecting succinate metabolism attenuates circulating succinate peaks, thereby reducing ROS production, inflammation, and tissue injury	Preclinical	Taghavi et al. (2022)
DMF	Rodent models (EAE mice, oxidative-stress rats) and RRMS human cohorts		Activation of the KEAP1-NRF2 antioxidant transcriptional program mitigates inflammation and oxidative stress; however, in fumarate-related pathologies, it may exert bidirectional and dose-dependent effects that warrant caution	Clinical approved	Bresciani et al. (2023)
Oral α-KG	Anti-aging mouse models and human RCTs	α-KG	Oral supplementation with α-ketoglutarate restores metabolic and epigenetic homeostasis by influencing DNA demethylases, mTOR signaling, and energy metabolism, thereby improving inflammation, muscle function, and metabolic parameters	Preclinical	Sandalova et al. (2023)
Exogenous ketone	Mouse colitis and metabolic-inflammation models; early-phase human studies	β-HB	Rapid elevation of circulating β-HB inhibits NLRP3 inflammasome activation, enhances mitochondrial function, and provides an alternative energy substrate, serving as a short-term pharmacological or nutritional intervention	Preclinical	Mohammed et al. (2024)
Ketogenic diet	Murine and human metabolic/neuroinflammatory models		Reducing carbohydrate intake and increasing endogenous ketone β-HB production suppress NLRP3 activation and improve mitochondrial and metabolic homeostasis	Preclinical	Neudorf and Little (2024)
GNE-140	Murine tumor models (syngeneic/xenograft) and co-culture tumor–immune assays	lactic acid	Inhibition of LDH reduces lactate production, redistributes glucose availability in the tumor microenvironment, enhances T-cell metabolism and effector function, and improves responsiveness to immune checkpoint blockade (anti-PD-1/PD-L1)	Preclinical	Verma et al. (2024)
AZD3965	Preclinical tumor models (mouse xenografts) and human phase I trials (advanced cancer)		Selective inhibition of the monocarboxylate transporter MCT1 blocks lactate transmembrane transport, leading to intratumoral lactate accumulation, microenvironmental acidification, and metabolic vulnerability, thus supporting immuno-metabolic therapeutic strategies	Clinical phase I	Halford et al. (2023)
Mito TEMPO	Cellular and murine models (organ injury, NASH, sepsis)	mtROS	Mitochondria-targeted SOD mimetics (superoxide scavengers) reduce mtROS, suppress oxidative stress, and preserve mitochondrial function and organ homeostasis	Preclinical	Wang et al. (2022a)
Elamipretide	Rodent mitochondrial dysfunction models; human trials (heart failure, myopathy)		Mitochondria-stabilizing peptides targeting cardiolipin or mitochondrial membranes improve coupling efficiency and energy production while reducing mtROS release and apoptosis/pyroptosis	Clinical phase II/III	Sabbah et al. (2025)
VENT-03	Healthy volunteer; planned SLE patient cohorts	mtDNA	Orally active small molecules directly inhibit cGAS, blocking mtDNA recognition and cGAMP synthesis to interrupt the upstream amplification of type I interferon signaling and alleviate mtDNA-driven inflammation	Clinical phase I	Pike et al. (2024)
Anifrolumab	Moderate-to-severe SLE patients		Anti-IFNAR1 monoclonal antibodies block type I interferon receptor signaling, suppress IFN-I-mediated pathogenic gene expression, and reduce disease activity	Clinical phase III	Morand et al. (2020)
Dapansutrile	Murine chronic inflammation, gout, and heart-failure models; human phase I–II trials		Orally active selective inhibitors of NLRP3 inflammasome assembly or activation reduce caspase-1 and IL-1β release, thereby mitigating inflammation-associated tissue damage	Clinical phase I–II	Wohlford et al. (2020)

Abbreviations: fibroblast growth factor 21; FGF21, growth differentiation factor 15; GDF15, α-ketoglutarate; α-KG, β-hydroxybutyrate; β-HB, mitochondrial reactive oxygen species; mtROS, mitochondrial DNA; mtDNA, nonalcoholic steatohepatitis; NASH, experimental autoimmune encephalomyelitis; EAE, relapsing-remitting multiple sclerosis; RRMS, randomized controlled trails; RCTs, systemic lupus erythematosus; SLE, glucagon-like peptide 1 receptor; GLP-1R, glucagon-like peptide-1; GLP-1, fibroblast growth factor receptor; FGFR, Klotho β; KLB, cytotoxic t lymphocytes; CD8^+^ T-cell, eukaryotic translation initiation factor 2α; eIF2α, C/EBP-homologous protein; CHOP, succinate receptor 1; SUCNR1, reactive oxygen species; ROS, cyclic adenosine monophosphate; cAMP, protein kinase A; PKA, camp response element-binding protein; CREB, kruppel-like factor 10; Klf10, formin homology 1 domain-containing protein; FH1, fumarate hydratase; FH, kelch-like ech-associated protein 1-nuclear factor erythroid 2-related factor 2; KEAP1-NRF2, mammalian target of rapamycin; mTOR, nucleotide-binding domain; leucine-rich repeat and pyrin domain-containing 3; NLRP3, lactate dehydrogenase; LDH, programmed cell death protein 1; PD-1, programmed death-ligand 1; PD-L1, monocarboxylate transporter 1; MCT1, superoxide dismutase; SOD, cyclic GMP-AMP; synthase; cGAS, cyclic GMP-AMP; cGAMP, interferon alpha/beta receptor subunit 1; IFNAR1, interferon type I; IFN-I, interleukin-1; beta; IL-1β.

Although hepatic mitochondria are increasingly recognized as central coordinators of aging-related diseases (Shin et al., 2023), several critical questions remain unresolved. We still have a very limited understanding of the initiating mechanisms underlying hepatic mitochondrial signaling. For instance, under aging-related or other stress conditions, how do mitochondria encode the duration and intensity of stress into distinct mitokines? What are the thresholds that separate adaptive responses from deleterious effects induced by these factors? Could transient mitochondrial perturbations leave lasting chromosomal or epigenetic marks that subsequently influence the genotype? An even more critical question is how mitochondrial-derived signals mediate precise inter-organ communication. Are there intrinsic positive or negative feedback loops, and might there be as-yet-undiscovered mediators of inter-tissue crosstalk? Addressing these unresolved questions is essential to resolve a core mechanistic uncertainty and to fully understand the contribution of mitochondria to organismal aging and aging-related diseases.

The next frontier lies in decoding the spatiotemporal logic of hepatic mitochondrial signaling, specifically how transient metabolic stress and chronic injury are transduced into distinct systemic messages through mitokines, metabolites, and ox-mtDNA. Resolving these temporal and quantitative dimensions will be crucial for distinguishing adaptive hormetic signaling from pathological remodeling that accelerates aging and aging-related diseases. Although this review emphasizes hepatic mitochondria as a central signaling hub, growing evidence shows that signals from skeletal muscle, the gut microbiota, and immune cells actively feedback to shape hepatic mitochondrial function and output, forming bidirectional and reinforcing inter-organ communication. Accordingly, future models should take this reciprocal regulation into account to avoid oversimplifying how organs communicate with one another. More importantly, future studies may also prioritize the development of multi-target combination therapies. For example, simultaneous modulation of FGF21 and GDF15 could provide synergistic benefits in the control of inflammation and metabolic dysfunction. In parallel, combination strategies acting on the same target represent another promising avenue. For example, distinct pharmacological agents such as metformin and halofuginone converge on the hepatic eIF2α-ATF4 pathway to induce GDF15, highlighting the feasibility of coordinated modulation of a single mitokine to improve metabolic outcomes. Moreover, greater emphasis should be placed on personalized medicine. Future interventions should be tailored according to individual genotypes, phenotypes, and hepatic mitochondrial factor profiles. Achieving this requires the establishment of dynamic monitoring systems for inter-organ mitochondrial communication, which would also advance the development of precision therapeutics. Ultimately, integrating preventive interventions with pharmacological reprogramming aims to translate hepatic mitochondrial signaling modulation into a broad-spectrum diagnostic and therapeutic strategy to delay aging and enhance quality of life.
